# A legend in one's own mind: The link between ambition and leadership evaluations

**DOI:** 10.1093/pnasnexus/pgae295

**Published:** 2024-08-20

**Authors:** Shilaan Alzahawi, Emily S Reit, Francis J Flynn

**Affiliations:** Graduate School of Business, Stanford University, 655 Knight Way, Stanford, CA 94305, USA; Graduate School of Business, Stanford University, 655 Knight Way, Stanford, CA 94305, USA; Graduate School of Business, Stanford University, 655 Knight Way, Stanford, CA 94305, USA

**Keywords:** ambition, leadership, leader effectiveness, leader evaluations, self-other discrepancies

## Abstract

Individuals who have more ambition—a persistent striving for success, attainment, and accomplishment—are more likely to become leaders. But are these ambitious individuals also more *effective* in leadership roles? We hypothesize that leader ambition is related to positive self-views of leader effectiveness that remain uncorroborated by relevant third-party actors. In a multiwave, preregistered study, we find evidence for this hypothesis using a sample of executives (*N* = 3,830 ratings of 472 leaders) who were rated by peers, subordinates, managers, and themselves on ten leadership competencies, including their ability to motivate others, manage collaborative work, coach and develop people, and present and communicate ideas. We consider the implications of our findings for both scholars and practitioners interested in leadership selection and development.

**Significance Statement**

Using third-party evaluations of a leader's effectiveness captured in actual 360-degree assessments, we find that more ambitious individuals are judged as no more effective in a leadership role than their less ambitious peers. Nonetheless, more ambitious individuals hold more positive views of their own effectiveness and are more likely to pursue leadership roles. This discrepancy suggests that society may need an alternative approach to leadership development and selection. Leader recruitment is often “passive,” waiting for applications to be submitted by aspiring leaders. Our findings show that the underlying premise—that individuals with the greatest leadership potential will self-select into leadership roles—may be unfounded. Rather than allowing ambitious individuals to dominate candidate pools for leadership opportunities, researchers and practitioners should consider ways to actively identify individuals who may be fully capable, but not fully confident, leaders.

## Introduction

Research has identified the importance of leadership in every domain of human activity, including, but not limited to, humanitarian aid provision (1), disaster prevention (2), conflict resolution (3), military training (4), safety enforcement (5), service delivery (6, 7), the healthcare sector (8, 9), higher education (10), financial institutions (6, 11, 12), government agencies (13), retail organizations (14, 15), research and development (16, 17), high-tech innovation (18), and manufacturing teams (19, 20). This work suggests that the right leader can make all the difference. But are those who aspire to become leaders necessarily the best candidates for the job? Or are they driven not by aptitude, but by ambition—a “persistent and generalized striving for success, attainment, and accomplishment” (21: p. 759)?

Given that most leader selection processes rely on self-selection into a candidate pool (i.e. individuals actively choose to be considered for leadership roles) (22), ambition may be a critical (albeit understudied) factor in leader selection and effectiveness (23). One may hope that the individuals who step forward to be considered for important leadership positions are the cream of the crop (i.e. that people are good at self-selection). However, this belief remains untested. Leadership scholars cannot say whether ambition is or is not, on balance, a positive indicator of leadership effectiveness.

Ambition corresponds to agency (24), which is considered an essential, if not the quintessential, leadership trait (25). Given that ambition represents a personal motive, feelings of ambition can be self-reported; that is, people are ambitious when they describe themselves as ambitious (26). Highly ambitious people possess a keen desire to take charge, assume greater responsibility, and guide others (23, 27). Their ambition leads them to obtain higher levels of education at more prestigious institutions (21, 28–30). More ambitious graduates then acquire more prestigious jobs (21), work more hours and are more connected to their work after hours (30, 31), and earn higher ranking positions with more lucrative salaries (21, 28, 32). In other words, more ambitious individuals are more likely to occupy high-status leadership roles. But are they also more likely to be *effective* in these leadership roles?

Studies of leader emergence and effectiveness suggest there may be a discrepancy between the attributes that help individuals attain leadership positions (referred to as “leadership prototypes” or “implicit leadership theories”) (33), on the one hand, and the attributes that help them be effective leaders, on the other (34, 35). According to this view, leader emergence and selection may be based on characteristics that *seem* to matter for leadership but are, in fact, “‘illusory correlations,’ abstract construals, or stereotypical proxies for leadership effectiveness” (36: p. 610). Indeed, many leaders fail due to, in part, the disconnect between the characteristics used for selection and those needed to address organizational challenges (37, 38). For example, facial appearance (“looking like a leader”) (36, 39) and vocal delivery (“sounding like a leader”) (40) predict leader emergence and selection, but not effectiveness.

We posit that ambition, while clearly predictive of leader emergence and (self-)selection^a^, may be unrelated to third-party ratings of leader effectiveness. Research on self-perception and self-awareness, particularly as these relate to one's own competence and skills, suggests that people struggle to gauge their own effectiveness and consistently report uncorroborated views of their competence (41). Individuals often regard themselves more positively than they deserve (referred to as the “above-average effect”) (42, 43)—and, in some cases, more negatively than they deserve (referred to as the “below-average effect”) (44, 45: p. 28). This suggests that even if people are motivated to form accurate appraisals of their own leadership effectiveness, it remains unclear whether they can.^b^

Instead, ambitious individuals may be drawn to leadership roles for reasons that are unrelated to their aptitude. Leadership roles provide material benefits (38), encouraging individuals to strive for ever-higher rungs on the corporate ladder. Not only do these high-ranking positions promise more money, they often offer a sweep of other benefits, such as generous 401(k) contributions, pension plans, and paid time off—all of which are highly desirable. Aside from financial incentives, leadership positions are also associated with psychological rewards, such as authority, freedom, social influence, and status (46, 47).

Ambitious individuals should be especially sensitive to the attractive rewards that come with leadership roles. Theories of ambition emphasize that ambitious individuals are driven by *extrinsic* success (e.g. status, rank, and wealth) rather than *intrinsic* success (e.g. well-being, competence, and job performance) (21). In other words, ambition “is marked by the desire for attainments independent of the degree to which obtaining these outcomes is based on superior performance” (21: p. 760). The allure of incentives associated with leadership roles, both material and psychological, may thus lead individuals to elevate their ambition beyond the limits of their aptitude (27).

When ambitious individuals strive to obtain leadership roles for reasons independent of their actual aptitude, how do they *justify* this striving? Decades of research on motivated reasoning (e.g. 48), cognitive dissonance (e.g. 49), and cognitive consistency (e.g. 50) suggest that ambitious individuals may inflate their self-perceptions of aptitude to justify their striving for career advancement and success. That is, ambition may lead individuals, regardless of third-party perceptions of their leadership aptitude, to both vie for leadership roles and hold more positive views of their effectiveness in those roles. Ultimately, we expect these forces to reduce self-other agreement in leadership effectiveness ratings (51, 52), by strengthening (weakening) the link between ambition and self-appraisals (third-party appraisals) of leader effectiveness.

We hypothesize that ambition is positively associated with self-ratings of leader effectiveness, but not associated with third-party ratings of leader effectiveness. In other words, we hypothesize a self-other discrepancy, in which ambition is calibrated to self-perceptions, but not to third-party perceptions, of leader effectiveness. To test these hypotheses, we relied on an archive of independently collected 360-degree leadership evaluations for a unique sample of executives who were rated by peers, subordinates, managers, and themselves (*N* = 3,830 ratings of 472 executives).

## Materials and methods

### Participants and procedure

We examine the relationship between ambition and leadership ratings using a sample of managers (*N* = 3,830 ratings of 472 leaders) enrolled in an executive education course offered by a West Coast business school in the United States. We asked each executive to rate their own personal ambition. To capture their rated effectiveness as a leader, we used an archive of leadership assessments independently gathered from each executive's current managers, peers, and subordinates, as well as themselves. This study received approval from an institutional review board (Stanford IRB-29451). Informed consent was obtained from all participants.

We collected data in two separate waves. To minimize common-method variance (particularly for testing the relationship between self-reported ambition and self-assessments of leader effectiveness), we ensured a significant time lag between our measures of ambition and effectiveness. The 360-degree leadership assessments from which we gathered effectiveness ratings and the survey in which we collected the ambition measure were separated by 27 days to over 4 months in the first wave of data collection and 1.5 to 2 years in the second wave of data collection.

Participants were individual managers enrolled in a 1-year leadership development program aimed at providing each participant with “the tools and techniques to become a more effective changemaker and leader.” As part of the curriculum, each manager was required to complete a 360-degree assessment of their leader effectiveness. To this end, each participant provided contact information for at least one manager, three peers, and three subordinates. In addition, the executives completed a self-assessment of their leader effectiveness. After completing the full 360-degree assessment, managers received a summary of their own and others’ evaluations. Data from participants’ leadership assessments were provided separately by the program administrator.

We sent each manager an invitation to take part in our study by completing a brief, online questionnaire in which we assessed their ambition. Participation was voluntary and had no effect on their standing in the program. We contacted a total of 1,290 executives, of whom 472 agreed to participate (37% response rate, $M_{\mathrm{age}}$ = 41.48, $SD_{\mathrm{age}}$ = 7.14, 32% female). Next, we matched these survey responses with the archive of leadership assessments completed by executives and their managers, peers, and subordinates. The matched sample containing both the survey responses and the 360-degree leadership assessments includes *N* = 3,830 ratings of 472 leaders.

### Measures

We report all measures collected. Across the two waves of data collection, we collected seven measures in total: ambition, motivation to lead (wave 1 only), drive to achieve (wave 2 only), leadership ability (wave 2 only), extraversion (wave 2, for exploratory purposes), gender, and age. Leadership ratings were obtained from an archival dataset provided by the leadership assessment program administrator.

#### Ambition

We assessed leader ambition using a seven-item, self-report measure (adapted from 28, 53). Participants rated their agreement with each of the following items: “My ultimate career aspiration is to be in a position of senior leadership,” “I have strong ambition when it comes to my career goals,” “I am highly motivated to get promoted quickly and often,” “I want my job to be highly respected by others,” “I am determined to have a highly successful career,” “It's important to me to attain a high-status position in my career,” and “I do NOT have a strong desire to advance far in my career” (reverse-scored). Responses were provided on a seven-point scale (1 = strongly disagree, 7 = strongly agree). We averaged the items to create a single measure of leader ambition (coefficient alpha $\hat{\alpha }$ = 0.83 and categorical omega $\hat{\omega_{C}}$ = 0.87) (54).

In addition to our main measure of leader ambition, we also collected a robustness check for ambition in each wave of data collection. In the first wave, we included motivation to lead as an additional measure of leader ambition. Specifically, we used Chan and Drasgow's (55) Affective-Identity subscale (coefficient alpha $\hat{\alpha }$ = 0.76 and categorical omega $\hat{\omega_{C}}$ = 0.82). The items were, “Most of the time, I prefer being a leader rather than a follower when working in a group,” “I am the type of person who is not interested to lead others” (reverse-scored), “I am definitely not a leader by nature” (reverse-scored), “I am the type of person who likes to be in charge of others,” “I believe I can contribute more to a group if I am a follower rather than a leader” (reverse-scored), “I usually want to be the leader in the groups that I work in,” “I am the type who would actively support a leader but prefers not to be appointed as leader” (reverse-scored), and “I have a tendency to take charge in most groups or teams that I work in.” We dropped one item (“I am seldom reluctant to be the leader of a group”) because it was negatively correlated with the total scale. In the second wave, we measured drive to achieve as an additional measure of leader ambition. Respondents rated themselves on drive to achieve, compared to the average person their age, on a five-point scale (highest 10%, above average, average, below average, lowest 10%).

#### Leader effectiveness

An institutional 360-degree leadership assessment measured 10 leadership competencies: managing self; decision making; growth orientation; accountability for results; managing collaborative work; motivating others; coaching and developing people; exerting influence and persuasion; presenting and communicating ideas; and handling conflict. Each competency was assessed with five items (for a total of 50) using a seven-point Likert-type scale ranging from 1 = not at all to 7 = always (coefficient alpha $\hat{\alpha }$ = 0.93 and coefficient omega $\hat{\omega }$ = 0.93 for self-ratings; coefficient alpha $\hat{\alpha }$ = 0.96 and categorical omega $\hat{\omega }$_C_ = 0.99 for third-party ratings). Two sample items for the motivation competency are “Motivates others to put forth greater effort” and “Recognizes others’ contributions.” In addition to our primary measure of self- and other-rated leader effectiveness obtained from the archival 360-degree assessments, we also included a robustness check for self-rated leader effectiveness in the second wave of data collection (results reported in the Supplemental Materials). Survey respondents rated themselves on their leadership ability, compared to the average person their age, on a five-point scale (highest 10%, above average, average, below average, lowest 10%).

### Analytic approach

To examine whether leader ambition is related to positive self-views that remain uncorroborated by relevant third-party actors, we ran two separate analyses. First, we ran frequentist mixed-effects models predicting effectiveness ratings from ambition (while including random intercepts for leaders and effectiveness items) and obtained the coefficient estimate ${\hat{\beta }}_{1}$ (and the 95% CI) for leader ambition. We ran these models separately for each of the five rater roles (self-ratings, all third-party ratings combined, managers, peers, and direct reports).

Second, we supply each of these results with Bayes factors to express the strength of the evidence (56). In contrast to the frequentist approach, Bayes factors allow us to quantify the relative evidence *in favor of the null hypothesis*. We ran two separate models to compute Bayes factors (using a default prior set by the BayesFactor package in R) (57): a full model and a null model. The specification of the full model is equivalent to the frequentist mixed-effects model described above: effectiveness ratings are predicted from ambition as well as random intercepts for leaders and effectiveness items. The null model is equivalent to the full model, with the single exception that it does not include a fixed effect for ambition (i.e. effectiveness ratings are predicted from the two random intercepts only).

We then calculated the Bayes factor in favor of the null model (BF_null_, defined as the likelihood of observing the data under the null model vs. the full/alternative model) and the Bayes factor in favor of the full/alternative model (where BF_alt_ = 1/BF_null_). For example, when BF_null_ = 3, the observed data are three times more likely under the null model (excluding ambition) than the alternative model (including ambition), which can be interpreted as *moderate* evidence for the null. Similarly, Bayes factors exceeding 10, 30, or 100 are interpreted as *strong*, *very strong*, or *extreme* evidence, respectively (56).

We conclude support for our hypothesis if (i) ambition is positively associated with self-rated leader effectiveness, as indicated by a positive and significant coefficient for ${\hat{\beta }}_{1}$, and (ii) ambition is not associated with third-party ratings of leader effectiveness, as indicated by BF_null_ > 1. For each analysis, we ran robustness checks to examine whether the same relationship holds for different measures of ambition (i.e. motivation to lead and drive to achieve) or a different measure of self-rated effectiveness (i.e. self-reported leadership ability, reported in the Supplemental Materials).

### Transparency and openness

Anonymized data, study materials, and analysis scripts are publicly accessible at https://osf.io/y8urj/. We describe our sampling plan, all data exclusions (if any), and all measures in the study. Both waves were preregistered. The preregistrations can be accessed at https://aspredicted.org/blind.php?x=yp66ej (wave 1) and https://osf.io/d2fv7 (wave 2). We deviated from our preregistrations in the following ways. First, rather than analyzing the two waves of data collection separately, we decided to pool the two samples and perform joint analyses on both waves. If we instead analyze the two samples separately, the results are highly similar and lead to the same qualitative conclusions (see Supplemental Materials). Second, for the first wave, we preregistered only a test for third-party perceptions, rather than a full test incorporating both self-views and third-party perceptions. Finally, for both waves, we had originally preregistered that we would run correlations on composite effectiveness scores. However, we later learned that a more appropriate modeling strategy would be to run mixed-effects models on the full, disaggregate data (i.e. all available ratings, rather than composites) to avoid loss of information and statistical power.

## Results

**Fig. 1.**
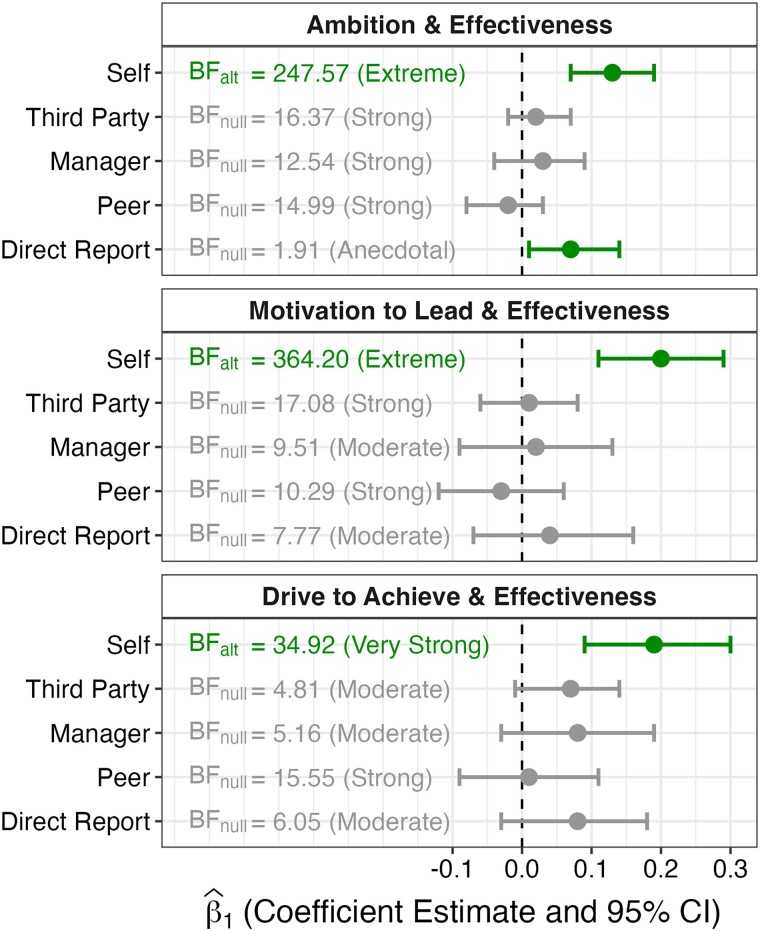
The relationship between ambition and leadership ratings for three measures of ambition and five rater roles. We ran mixed-effects models predicting effectiveness ratings from ambition as well as random intercepts for leaders and effectiveness items and obtained the coefficient estimates for ambition (${\hat{\beta }}_{1}$). The color of the bar indicates the significance of ${\hat{\beta }}_{1}$ (green bars indicate a rejection of the null; gray bars indicate a failure to reject the null). The Bayes factors express the strength of the evidence: BF_alt_ expresses the strength of the evidence for a full model (which includes a fixed effect for ambition), whereas BF_null_ expresses the strength of the evidence for a null model (which does not include a fixed effect for ambition). Note that BF_alt_ = 1/BF_null_. The color of the text indicates the direction of the evidence (green text indicates support for the full model; gray text indicates support for the null model).

Figure 1 displays the relationship between leader ambition and evaluations of overall leader effectiveness. We find extreme evidence (Bayes Factor > 100) (56) that leaders with higher ambition rate themselves as *more effective* overall in their leadership role, ${\hat{\beta }}_{1}$ = 0.13, 95% CI (0.07, 0.19), *P* < 0.001, extreme BF_alt_ = 247.57. However, we find strong evidence (10 < BF < 30) (56) that leaders with higher ambition are rated as *no more effective* overall in their leadership role by relevant third-party actors (i.e. their managers, peers, and direct reports combined), ${\hat{\beta }}_{1}$ = 0.02, 95% CI (−0.02, 0.07), *P* = 0.293, strong BF_null_ = 16.37, by their managers, ${\hat{\beta }}_{1}$ = 0.03, 95% CI (−0.04, 0.09), *P* = 0.425, strong BF_null_ = 12.54, or by their peers, ${\hat{\beta }}_{1}$ = −0.02, 95% CI (−0.08, 0.03), *P* = 0.407, strong BF_null_ = 14.99, and anecdotal evidence (1 < BF < 3) (56) that leaders with higher ambition are rated as *no more effective* overall in their leadership role by their direct reports, ${\hat{\beta }}_{1}$ = 0.07, 95% CI (0.01, 0.14), *P* = 0.031, anecdotal BF_null_ = 1.91.

We formally test the difference between the regression coefficients for self-assessments and third-party assessments of leader effectiveness (58). We find that the relationship between ambition and leader self-evaluations is significantly greater than the relationship between ambition and third-party leader evaluations, $\Delta_{\beta_{1}}$ = 0.10, 95% CI (0.04, 0.17), *P* = 0.003, manager evaluations, $\Delta_{\beta_{1}}$ = 0.10, 95% CI (0.03, 0.18), *P* = 0.013, and peer evaluations, $\Delta_{\beta_{1}}$ = 0.15, 95% CI (0.08, 0.22), *P* < 0.001. For direct report evaluations, we find a small but nonsignificant difference, $\Delta_{\beta_{1}}$ = 0.06, 95% CI (−0.02, 0.13), *P* = 0.108.

To further corroborate the discrepancy between self-evaluations and third-party evaluations of leader effectiveness, we ran two preregistered robustness checks using two alternative measures of leader ambition. We find extreme evidence that leaders with higher motivation to lead rate themselves as *more effective* overall in their leadership role, ${\hat{\beta }}_{1}$ = 0.20, 95% CI (0.11, 0.29), *P* < 0.001, extreme BF_alt_ = 364.20. Further corroborating the hypothesized self-other discrepancy, we find strong evidence that leaders with higher motivation to lead are rated as *no more effective* overall in their leadership role by all relevant third-party actors, ${\hat{\beta }}_{1}$ = 0.01, 95% CI (−0.06, 0.08), *P* = 0.738, strong BF_null_ = 17.08, or by their peers, ${\hat{\beta }}_{1}$ = −0.03, 95% CI (−0.12, 0.06), *P* = 0.457, strong BF_null_ = 10.29, and moderate evidence (3 < BF < 10) (56) that leaders with higher motivation to lead are rated as *no more effective* overall in their leadership role by their managers, ${\hat{\beta }}_{1}$ = 0.02, 95% CI (−0.09, 0.13), *P* = 0.694, moderate BF_null_ = 9.51, or by their direct reports, ${\hat{\beta }}_{1}$ = 0.04, 95% CI (−0.07, 0.16), *P* = 0.437, moderate BF_null_ = 7.77.

Again, we formally test the difference between the regression coefficients for self-assessments and third-party assessments of leader effectiveness. We find that the relationship between motivation to lead and leader self-evaluations is significantly greater than the relationship between motivation to lead and third-party leader evaluations, $\Delta_{\beta_{1}}$ = 0.18, 95% CI (0.09, 0.28), *P* < 0.001, manager evaluations, $\Delta_{\beta_{1}}$ = 0.17, 95% CI (0.05, 0.29), *P* = 0.009, peer evaluations, $\Delta_{\beta_{1}}$ = 0.23, 95% CI (0.12, 0.34), *P* < 0.001, and direct report evaluations, $\Delta_{\beta_{1}}$ = 0.15, 95% CI (0.03, 0.27), *P* = 0.020.

As our second preregistered robustness check, we assess whether the same self-other discrepancy holds for the relationship between drive to achieve and leadership ratings. We find very strong evidence (30 < BF < 100) (56) that leaders with a higher drive to achieve rate themselves as *more effective* overall in their leadership role, ${\hat{\beta }}_{1}$ = 0.19, 95% CI (0.09, 0.30), *P* < 0.001, very strong BF_alt_ = 34.92. Further corroborating our findings, we find strong evidence that leaders with a higher drive to achieve are rated as *no more effective* overall in their leadership role by their peers, ${\hat{\beta }}_{1}$ = 0.01, 95% CI (−0.09, 0.11), *P* = 0.800, strong BF_null_ = 15.55, and moderate evidence that leaders with a higher drive to achieve are rated as *no more effective* overall in their leadership role by all relevant third-party actors, ${\hat{\beta }}_{1}$ = 0.07, 95% CI (−0.01, 0.14), *P* = 0.078, moderate BF_null_ = 4.81, by their managers, ${\hat{\beta }}_{1}$ = 0.08, 95% CI (−0.03, 0.19), *P* = 0.152, moderate BF_null_ = 5.16, or by their direct reports, ${\hat{\beta }}_{1}$ = 0.08, 95% CI (−0.03, 0.18), *P* = 0.158, moderate BF_null_ = 6.05.

Again, we formally test the difference between the regression coefficients for self-assessments and third-party assessments of leader effectiveness. We find that the relationship between drive to achieve and leader self-evaluations is significantly greater than the relationship between drive to achieve and third-party leader evaluations, $\Delta_{\beta_{1}}$ = 0.12, 95% CI (0.02, 0.23), *P* = 0.030, and peer evaluations, $\Delta_{\beta_{1}}$ = 0.18, 95% CI (0.06, 0.30), *P* = 0.007. We find a small but nonsignificant difference for manager evaluations, $\Delta_{\beta_{1}}$ = 0.11, 95% CI (−0.01, 0.24), *P* = 0.067, and direct report evaluations, $\Delta_{\beta_{1}}$ = 0.12, 95% CI (−0.01, 0.24), *P* = 0.060.

### Exploratory analysis of separate leadership competencies

**Fig. 2.**
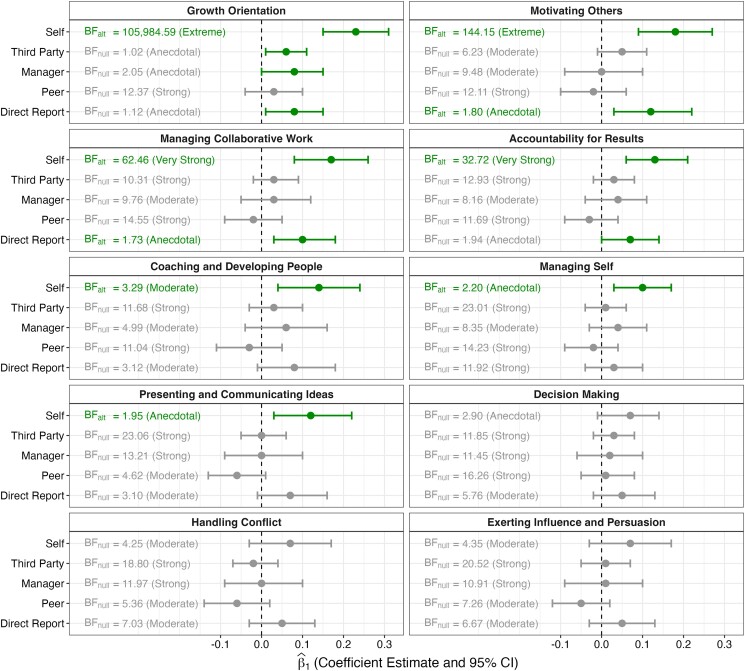
The relationship between ambition and leadership ratings for ten leadership competencies and five rater roles. We ran mixed-effects models predicting effectiveness ratings from ambition as well as random intercepts for leaders and effectiveness items and obtained the coefficient estimates for ambition (${\hat{\beta }}_{1}$). The color of the bar indicates the significance of ${\hat{\beta }}_{1}$ (green bars indicate a rejection of the null; gray bars indicate a failure to reject the null). The Bayes factors express the strength of the evidence: BF_alt_ expresses the strength of the evidence for a full model (which includes a fixed effect for ambition), whereas BF_null_ expresses the strength of the evidence for a null model (which does not include a fixed effect for ambition). Note that BF_alt_ = 1/BF_null_. The color of the text indicates the direction of the evidence (green text indicates support for the full model; gray text indicates support for the null model).

In addition to our main, preregistered analyses, we ran an exploratory analysis of the ten leadership competencies. Specifically, we assess the degree of self-other discrepancy that appears for each competency. Figure 2 displays the relationship between leader ambition and leader effectiveness for each separate competency.

First, while we find extreme evidence that leaders with higher ambition rate themselves as demonstrating greater growth orientation (e.g. soliciting developmental feedback to improve performance and asking for opportunities to further career development), ${\hat{\beta }}_{1}$ = 0.23, 95% CI (0.15, 0.31), *P* < 0.001, extreme BF_alt_ = 105,984.59, we find anecdotal to strong evidence that relevant third-party actors, managers, peers, and direct reports *do not* observe greater growth orientation in leaders with higher ambition (1.02 < BF_null_ < 12.37).

Second, while we find extreme evidence that leaders with higher ambition rate themselves as demonstrating greater ability to motivate others (e.g. understanding what motivates each person they work with and contributing to a strong sense of team identity), ${\hat{\beta }}_{1}$ = 0.18, 95% CI (0.09, 0.27), *P* < 0.001, extreme BF_alt_ = 144.15, we find moderate to strong evidence that relevant third-party actors, managers, and peers *do not* rate leaders with higher ambition as more effective at motivating others (6.23 < BF_null_ < 12.11). We do find, however, anecdotal evidence that direct reports rate leaders with higher ambition as more effective at motivating others, ${\hat{\beta }}_{1}$ = 0.12, 95% CI (0.03, 0.22), *P* = 0.012, anecdotal BF_alt_ = 1.80.

Third, while we find very strong evidence that leaders with higher ambition rate themselves as demonstrating greater ability to manage collaborative work (e.g. following up on incomplete tasks and getting buy-in for processes that involve multiple people), ${\hat{\beta }}_{1}$ = 0.17, 95% CI (0.08, 0.26), *P* < 0.001, very strong BF_alt_ = 62.46, we find moderate to strong evidence that relevant third-party actors, managers, and peers *do not* observe greater ability to manage collaborative work in leaders with higher ambition (9.76 < BF_null_ < 14.55). We do find, however, anecdotal evidence that direct reports observe greater ability to manage collaborative work in leaders with higher ambition, ${\hat{\beta }}_{1}$ = 0.10, 95% CI (0.03, 0.18), *P* = 0.009, anecdotal BF_alt_ = 1.73.

Fourth, while we find very strong evidence that leaders with higher ambition rate themselves as demonstrating greater accountability for results (e.g. taking projects to completion and accepting responsibility for mistakes), ${\hat{\beta }}_{1}$ = 0.13, 95% CI (0.06, 0.21), *P* < 0.001, very strong BF_alt_ = 32.72, we find anecdotal to strong evidence that relevant third-party actors, managers, peers, and direct reports *do not* observe greater accountability for results in leaders with higher ambition (1.94 < BF_null_ < 12.93).

Fifth, while we find moderate evidence that leaders with higher ambition rate themselves as demonstrating greater ability to coach and develop people (e.g. investing time and energy to coach others and providing development opportunities), ${\hat{\beta }}_{1}$ = 0.14, 95% CI (0.04, 0.24), *P* = 0.008, moderate BF_alt_ = 3.29, we find moderate to strong evidence that relevant third-party actors, managers, peers, and direct reports *do not* observe greater ability to coach and develop people in leaders with higher ambition (3.12 < BF_null_ < 11.68).

Finally, while we find anecdotal evidence that leaders with higher ambition rate themselves as demonstrating greater ability to self-manage (e.g. maintaining a positive attitude and recovering quickly from setbacks), ${\hat{\beta }}_{1}$ = 0.10, 95% CI (0.03, 0.17), *P* = 0.008, anecdotal BF_alt_ = 2.20, and present and communicate ideas (e.g. presenting complex material in a way that is clear and engaging and reinforcing messages with specific takeaways or action items), ${\hat{\beta }}_{1}$ = 0.12, 95% CI (0.03, 0.22), *P* = 0.012, anecdotal BF_alt_ = 1.95, we find moderate to strong evidence that relevant third-party actors, managers, peers, and direct reports *do not* observe greater ability to self-manage (8.35 < BF_null_ < 23.01) or present and communicate ideas (3.10 < BF_null_ < 23.06) in leaders with higher ambition.

For three of the ten leadership competencies, we find anecdotal to moderate evidence against a relationship between leader ambition and self-evaluations of leadership effectiveness. Leaders with higher ambition *do not* rate themselves as demonstrating significantly greater decision-making ability, ${\hat{\beta }}_{1}$ = 0.07, 95% CI (−0.01, 0.14), *P* = 0.075, anecdotal BF_null_ = 2.90, conflict resolution skills, ${\hat{\beta }}_{1}$ = 0.07, 95% CI (−0.03, 0.17), *P* = 0.154, moderate BF_null_ = 4.25, or influence and persuasion, ${\hat{\beta }}_{1}$ = 0.07, 95% CI (−0.03, 0.17), *P* = 0.177, moderate BF_null_ = 4.35. Similarly, relevant third-party actors, managers, peers, and direct reports *do not* observe greater decision-making ability (5.76 < BF_null_ < 16.26), conflict resolution skills (5.36 < BF_null_ < 18.80), or ability to exert influence and persuasion (6.67 < BF_null_ < 20.52) in leaders with higher ambition.

Overall, the exploratory analyses demonstrate discrepant perceptions of effectiveness on seven out of ten leadership competencies, including the extent to which ambitious leaders are perceived by themselves vs. others as having a growth orientation; motivating others; managing collaborative work; being accountable for results; coaching and developing people; managing themselves; and presenting and communicating ideas. These discrepancies seem especially prominent among managerial and peer ratings. Direct reports, however, appear to show slightly greater sensitivity to the ambition levels of their leaders. Indeed, we find anecdotal evidence that direct reports perceive ambitious leaders as better able to motivate others and manage collaborative work.^c^

## Discussion

The concept of ambition—striving for higher levels of power and status in one's career—receives ample attention from the popular press and lay people alike (59, 60). Yet, despite its widespread presence in public discourse, ambition has received surprisingly little scholarly attention. We answer a call for more research on the potential link between ambition and leader effectiveness (21, 61) by studying whether it is well-calibrated to third-party perceptions (i.e. whether more ambitious people are rated as more effective leaders). Our results suggest that people do not calibrate their personal ambition according to their aptitude. Instead, we find consistent evidence that ambition is related to favorable self-perceptions, but not to favorable third-party perceptions, of leader effectiveness.

We further investigate the found null relationship between ambition and third-party evaluations of leader effectiveness in two supplemental studies reported in the Supplemental Online Materials. These two supplemental studies find that the null relationship between ambition and third-party ratings of effectiveness replicates when we rely on different settings (i.e. a leadership simulation and a group decision-making task); different samples (i.e. MBA students and a nationally representative sample); different raters (i.e. expert, C-suite judges and team members); different measures of effectiveness (e.g. empowering leader behavior and a behavioral measure of “information surfacing”); different measures of ambition (e.g. status motives); and additional controls (i.e. the “Big Five” personality traits). Notably, the null relationship replicates in a study in which we circumvented potential selection effects by relying on a nationally representative sample with random assignment of a leadership role.

Despite this missing link between ambition and third-party ratings of leader effectiveness, ambitious individuals are more likely to occupy high-status leadership roles. This striking finding is in line with prior theorizing on the effectiveness-emergence paradox (62), leader over-emergence and leader under-emergence (35, 63), the ascription–actuality trait theory of leadership (34), and the ascription–achievement framework (64). Similar gaps between leader emergence and leader effectiveness have been demonstrated for a wide range of characteristics, including facial appearance (36, 39, 65, 66), vocal delivery (40), gender and masculinity (67), dominance and overconfidence (68), narcissism (69, 70), and performance in nonleadership roles (71).

A potential explanation for the found emergence–effectiveness gap among ambitious leaders can be drawn from leader distance theory (72, 73): ambition may be a trait that is beneficial in the “emerging zone” (i.e. in short-term contexts involving unacquainted individuals and early-stage relationships, such as hiring situations) but inconsequential in the “enduring zone” (i.e. in long-term contexts involving acquainted individuals and continuing relationships, such as employment situations).

In short-term contexts (i.e. in the “emerging zone”), a target's actual or potential leader effectiveness is often unknown to observers, and observers thus need to rely on *signals* to gauge an individual's (potential) effectiveness (74, 75). One such signal could be the ambition levels expressed by targets: ambition shares conceptual overlap with several qualities that are considered prototypical of leaders, including dedication (e.g. motivation, hard work, determination, and goal-orientedness) (76–79), conscientiousness (80, 81), achievement striving (80, 82), and agency (24). Supporting this theorizing, we find (see Supplemental Online Materials) that lay individuals *predict* that ambitious targets will be more effective leaders.

However, as opportunities to observe ambitious leaders increase (i.e. in the “enduring zone”), ratings of effectiveness will increasingly be based on actual leadership behaviors (72, 73). These behavioral displays of leadership may be no more effective for ambitious leaders because, as theorized earlier, ambition draws people to leadership roles for reasons unrelated to their effectiveness. Thus, while implicit theories might lead third-party actors to have positively inflated *first impressions* of ambitious individuals (and, consequently, to be more likely to hire these individuals into leadership roles), increased visibility of actual leadership behaviors may lead to more tempered, and perhaps more realistic, ratings of leadership effectiveness over time. We await a more direct test of this hypothesis, in which time and leader visibility are considered moderators of the ambition–effectiveness relationship.

Evidence against a relationship between ambition and third-party evaluations of leader effectiveness calls into question the approach taken by institutions that identify and train future leaders (e.g. business schools). Many of these institutions are “passive” in their recruitment, waiting for applications to be submitted by individuals who aspire to take on senior leadership roles. This passive approach presumes that individuals with the greatest leadership potential will apply, but this assumption may be unfounded. Perhaps these institutions are not selecting individuals with the greatest ability but those with the greatest *ambition*—a trait that is not indicative of superior leadership potential. Our findings should caution these institutions to de-emphasize ambition as a factor in admission by soliciting a wider, more representative pool of applicants (e.g. 22, 83). Doing so would help them deliver on their stated objective—to train the best future leaders.

Along a similar vein, our work offers useful advice for human resource managers who oversee internal labor markets in the workplace. Rather than allowing more ambitious employees to dominate the candidate pools for more senior leadership roles, human resource managers should recruit candidates based on individual traits that are known to predict leadership aptitude, such as intelligence (84) and prosociality (85). In addition, these managers may need to develop direct interventions aimed at fostering feelings of ambition in those employees who possess the greatest leadership potential. That is, they must identify and encourage high-potential leaders early in their career—to buoy their confidence—before those individuals with low ambition and high aptitude opt out of pursuing leadership opportunities.

To be clear, we do not suggest that high-potential individuals who lack a desire to lead should be forced into leadership roles. We find that ambition—which is marked by a desire for extrinsic outcomes such as power, status, rank, and wealth—is not related to effectiveness in a leadership role. However, prior work finds that individuals with an *intrinsic* motivation to lead—who are prosocial, collectively minded, and committed to the group's success rather than their own—are more effective (68, 85). Noting this, we suggest that those selecting leaders focus on individuals who are motivated *for the right reasons*.

Our study has some limitations that offer promising avenues for future research. First, we utilized subjective ratings of leader effectiveness provided by peers, subordinates, and managers. These ratings capture a specific, subjective form of leadership evaluation and might overlook a leader's effectiveness at meeting concrete goals and objective performance measures (e.g. key performance indicators). A future study might examine whether ambitious leaders are less liked (cf. 86), but more likely to get the job done. Highly assertive leaders, for example, often have poor social relationships, despite showing meaningful progress toward achieving instrumental goals (87).

Second, future research might identify potential boundary conditions of the relationship between ambition and third-party effectiveness ratings. One possible boundary condition is culture (88). We expect a stronger relationship between ambition and leader effectiveness in non-Western cultures for two reasons. First, in interdependent cultures, individuals are more concerned with maximizing group success than personal goals (89). Concern for the group may motivate people to self-regulate their ambition, even when presented with strong incentives to pursue high-ranking leadership roles. Second, compared with our US sample, some cultures exhibit less of a positivity bias (e.g. Japan, 90). Individuals from these cultures might be less likely to develop an inflated sense of leadership ambition because they receive more accurate feedback from others about their flaws and limitations.

Given the importance of leadership across all aspects of social life, understanding whether individuals who possess more ambition are also more capable to lead is of paramount importance. The present research offers a rigorous empirical examination of the relationship between ambition and leader effectiveness using an archive of actual 360-degree leadership evaluations. We find consistent evidence that more ambitious individuals rate themselves as more capable to lead than do their less ambitious peers. However, others disagree with that assessment. This discrepancy suggests that society may need an alternative approach to leadership development—not rewarding ambitious people with more leadership opportunities but finding ways to instill ambition in people who possess more leadership aptitude.

## Supplementary Material

pgae295_Supplementary_Data

## Data Availability

Anonymized data, study materials, and analysis scripts are publicly accessible at https://osf.io/y8urj/ (91). This study was run in two preregistered waves. Wave 1: https://aspredicted.org/blind.php?x=yp66ej and wave 2: https://osf.io/d2fv7.

## Notes

a In two pilot studies (described in the Supplemental Materials), we find evidence that lay theories about leadership favor the highly ambitious and that ambitious individuals are more likely to emerge as leaders. In a pilot using a simple vignette describing someone with high or low ambition (Pilot 1), ambitious targets were rated as having greater leadership aptitude, ΔM = 2.63, *t*(149) = 14.33, *P* < 0.001. In a time-lagged study of college students in the United States (Pilot 2), ambitious freshmen were more likely to emerge as leaders by joining student government during their time in college, $\hat{\beta }$ = 0.32, 95% CI (0.30, 0.33), *z* = 40.26, *P* < 0.001.

b In a pilot study (Pilot 2, described in the Supplemental Materials), we find suggestive evidence—among *N* = 14,235,487 college freshmen at 1,840 institutions in the United States and *n* = 527,159 college seniors at 722 institutions in the United States—that the “above-average effect” is more pronounced among individuals with greater ambition. For example, the proportion of above-average beliefs in freshmen year of college increases from 8% (for students with the lowest ambition) to 78% (for students with the highest ambition), $\hat{\beta }$ = 0.92, 95% CI (0.92, 0.92), *z* = 999.81, *P* < 0.001. In other words, 78% of highly ambitious freshmen believe they are above-average in the domain of leadership ability.

c In our other, preregistered analyses and robustness checks (reported in the Supplemental Online Materials), these findings do not appear to replicate, i.e. we find moderate evidence *against* a relationship between *motivation to lead* and direct report evaluations of overall effectiveness (moderate BF_null_ = 7.77); ability to motivate others (moderate BF_null_ = 7.11); and ability to manage collaborative work (moderate BF_null_ = 8.98) and moderate to strong evidence *against* a relationship between *drive to achieve* and direct report evaluations of overall effectiveness (moderate BF_null_ = 6.05); ability to motivate others (moderate BF_null_ = 3.28); and ability to manage collaborative work (strong BF_null_ = 12.72).
