# Supplementary material for: A legend in one's own mind: The link between ambition and leadership evaluations
Source: PNAS Nexus. 2024 Aug 20;3(8):pgae295. doi: 10.1093/pnasnexus/pgae295 (PMC11333923; doi:10.1093/pnasnexus/pgae295)
Supplement: pgae295_Supplementary_Data [file pgae295_supplementary_data.pdf]

Supplemental Online Materials for

A Legend in One's Own Mind: The Link Between Ambition and Leadership Evaluations

Shilaan Alzahawi[1], Emily S. Reit[1], & Francis J. Flynn[1]

[1] Graduate School of Business, Stanford University, Stanford, California, United States

Correspondence should be addressed to Shilaan Alzahawi, 655 Knight Way, Stanford, CA 94305.

Email: shilaan@stanford.edu

Author Contributions:
Conceptualization: S. Alzahawi, E.S. Reit, and F.J. Flynn;
Methodology: S. Alzahawi, E.S. Reit, and F.J. Flynn;
Software: S. Alzahawi and E.S. Reit;
Validation: S. Alzahawi;
Formal Analysis: S. Alzahawi and E.S. Reit;
Resources: F.J. Flynn;
Data Curation: S. Alzahawi and E.S. Reit;
Writing – Original Draft: S. Alzahawi, E.S. Reit, and F.J. Flynn;
Writing – Review & Editing: S. Alzahawi and F.J. Flynn;
Visualization: S. Alzahawi

Competing Interest Statement: The authors declare no competing interests.

Funding: The authors declare no funding.

Classification: Psychological and Cognitive Sciences (Social and Political Sciences)

Keywords: ambition, leadership, leader effectiveness, leader evaluations, self-other discrepancies

This PDF file includes:

Main Text
Figures S1 to S9
Tables S1 to S6

Author note:

Anonymized data, study materials, and analysis scripts can be found at https://osf.io/y8urj.

This study was run in two preregistered waves. We also preregistered Pilot S2 and Supplemental S2.

Wave 1: https://aspredicted.org/blind.php?x=yp66ej
Wave 2: https://osf.io/d2fv7/?view_only=02aa25f66bbb4df89e95073346ec59b9
Pilot S2: https://osf.io/btynm?view_only=af7f64e52c37443ca62103b44c78cec4
Supplemental S2: https://aspredicted.org/blind.php?x=9vj8ua

Main Text

In this Supplement, we run several preregistered analyses not reported in the main paper. In the main paper, we pooled our two samples and performed joint analyses on both waves. Here, we show that the results are highly similar and lead to the same qualitative conclusions when we instead follow our preregistered plan to separately analyze the two waves of data collection.

Participants and Procedure

The samples in Wave 1 and Wave 2 were non-overlapping: we contacted executives from different cohorts of the leadership development program. For Wave 1, we contacted a total of 288 executives, of whom 181 agreed to participate (63% response rate). Due to missing ratings for two participants, the final sample included 179 executives (M_{age} = 40.58, SD_{age} = 7.31, 36% female). For Wave 2, we contacted a total of 1,002 executives, and obtained 294 responses (29% response rate). We removed one duplicate observation from an executive who filled in the survey twice, leading to a final sample of 293 executives (M_{age} = 42.09, SD_{age} = 6.98, 29% female).

After matching the executives with the archive of leadership assessments completed by the executives and their managers, peers, and subordinates, our final sample included *N* = 1,386 ratings (*n* = 179 self-reports and *n* = 1,207 third-party assessments) in Wave 1 and *N* = 2,444 ratings (*n* = 293 self-reports and *n* = 2,151 third-party assessments) in Wave 2.

Ambition Scale

To create our ambition measure, we adapted the "ambition value" scale from Ashby and Schoon (1), which was developed to capture ambition among adolescents. We took the first item of the two-item scale and rephrased it from "It matters to me to get promotion so I can get ahead" to "I am highly motivated to get promoted quickly and often" for clarity. We dropped the second item ("It matters to me to get a job with a real challenge") because it appears to focus on intrinsic motivation rather than ambition. To strengthen this scale, we added three items of the 6-item "Status Motive" scale from Neel and colleagues (2): "It's important to me that other people look up to me", "I want to be in a position of leadership", and "It's important to me that others respect my rank or position." To clearly reflect a striving for career advancement and success, we rephrased these items as follows: "I want my career to be highly respected by others", "My ultimate career goal is to be in a position of senior leadership", and "It's important to me to attain a high-status position in my career." We did not include the last three items of the Status Motive scale ("I do things to ensure that I don't lose the status I have", "I do not like being at the bottom of a hierarchy", and "I do not worry very much about losing status"), because these items focus on status loss.

Finally, we added a reverse-coded item ("I do NOT have a strong desire to advance in my career") for attentiveness and two similarly phrased items ("I have strong ambition when it comes to my career goals" and "I am determined to have a highly successful career") for consistency.

Prior Sensitivity Analysis

We rely on Bayesian mixed effects models in both the main and supplemental analyses. We ran Bayesian mixed effects models using the BayesFactor package in R (3). The prior assumes the standardized slope is normally distributed with $\beta \sim N(0, g\sigma^2(X'X)^{-1})$, where g is Inverse Gamma distributed, $g \sim IG(1/2, r/2)$, and the default ("medium") prior sets $r = \sqrt{2}/4$, the wide prior sets $r = 1/2$, and the ultrawide prior sets $r = \sqrt{2}/2$.

We note that this approach represents a conservative test. When using Bayes Factors, "evidence accumulates more slowly for the null hypothesis than for the alternative hypothesis" (4: p. 12). Concluding support for the null requires many observations in which the effect size is close to zero. Thus, if the observed Bayes factors in favor of the null satisfy our minimum threshold, we will have passed a relatively high bar for judging the strength of empirical evidence (i.e., a substantially higher bar than is required to conclude support for the alternative hypothesis).

In all Bayesian analyses in the main paper, we rely on the default ("medium") prior, assuming that small effects are likelier than large ones. We do this for two reasons. First, effects documented in behavioral research tend to be small (5, 6). Meta-analytic findings offer a sense of the effect size that we may expect in research on the (personality trait) correlates of leadership effectiveness: e.g., leadership evaluations exhibit correlations of r = .31 and r = –.24 with extraversion and neuroticism, respectively (7). Our chosen prior reflects these past findings.

Second, our prior represents a high threshold for the test of our null hypothesis. Priors affect the likelihood of finding support for the null hypothesis; the smaller (i.e., less "wide") the prior, the less likely it becomes to find (spurious) support. This phenomenon occurs because the prior specifies the effect size expected under the *alternative* hypothesis. When larger effect sizes are expected under the alternative, observations with smaller effect sizes are more surprising and lead to a quicker rejection of the alternative. In contrast, when we expect smaller effect sizes under the alternative, it takes many (close to) null observations before we can reject the alternative and conclude having obtained evidence for the null. By using priors that assume relatively small effects under the alternative hypothesis, our test of the null hypothesis becomes more severe, safeguarding us against potential false negatives.

In sum, our approach entails a stringent test of the null hypothesis for two main reasons. First, using Bayes factors, it is harder to obtain evidence in favor of a null hypothesis than an alternative hypothesis: the former requires more hypothesis-consistent observations than the latter (4). Second, it is harder to obtain evidence in favor of a null when using priors that assume relatively small effects. Thus, passing our threshold to support the null would allow us to infer the lack of a relationship between ambition and leadership effectiveness with a relatively high degree of confidence. Nonetheless, we do various sensitivity analyses to show that our results are robust to the choice of priors in all Bayesian tests.

A robustness check is displayed in Figure S1, where we show the robustness of the results across many possible prior specifications (ranging from 1/3 times to 3 times the width of the default prior). We find evidence in favor of our hypotheses across the full range of prior distributions.

When relying on a wide prior, we find extreme evidence *for* a relationship between ambition and self-evaluations of overall effectiveness in the leadership role, extreme BF_{alt} = 182.20, as well as strong evidence *against* a relationship between ambition and third-party evaluations of overall effectiveness in the leadership role, strong BF_{null} = 23.11. In addition, we find extreme evidence *for* a relationship between motivation to lead and self-evaluations of overall effectiveness in the leadership role, extreme BF_{alt} = 280.78, as well as strong evidence *against* a relationship between motivation to lead and third-party evaluations of overall effectiveness in the leadership role, strong BF_{null} = 24.07.

When relying on an ultrawide prior, we find extreme evidence *for* a relationship between ambition and self-evaluations of overall effectiveness in the leadership role, extreme BF_{alt} = 131.40, as well as very strong evidence *against* a relationship between ambition and third-party evaluations of overall effectiveness in the leadership role, very strong BF_{null} = 32.65. In addition, we find extreme evidence *for* a relationship between motivation to lead and self-evaluations of overall effectiveness in the leadership role, extreme BF_{alt} = 208.15, as well as very strong evidence *against* a relationship between motivation to lead and third-party evaluations of overall effectiveness in the leadership role, very strong BF_{null} = 33.99.

Overall, the results shown in Figure S1 suggest that our analyses are robust to the specification of the prior distribution. Across the entire range of prior distributions, we find evidence *for* a relationship between ambition and self-evaluations of overall effectiveness in the leadership role and evidence *against* a relationship between ambition and third-party evaluations of overall effectiveness in the leadership role.

Results by Wave

Tables S1 and S2 report basic descriptive statistics and correlations for the first and second wave of data collection, respectively. Figure S2 displays the relationship between leader ambition and evaluations of overall effectiveness in the leadership role, split by data collection wave.

In the first wave of data collection, we find moderate evidence (3 < BF < 10) (8) that leaders with higher levels of ambition rate themselves as *more effective* overall in their leadership role, $\hat{\beta}_1$ = 0.14, 95% CI [0.05, 0.24], *p* = .005, moderate BF_{alt} = 4.00. However, we find strong evidence (10 < BF < 30) (8) that leaders with higher levels of ambition are rated as *no more effective* overall in their leadership role by their peers, $\hat{\beta}_1$ = 0.01, 95% CI [-0.09, 0.10], *p* = .907, strong BF_{null} = 12.60, moderate evidence that leaders with higher levels of ambition are rated as *no more effective* overall in their leadership role by relevant third-party actors (i.e., their managers, peers, and direct reports combined), $\hat{\beta}_1$ = 0.05, 95% CI [-0.02, 0.13], *p* = .146, moderate BF_{null} = 6.29 or by their managers, $\hat{\beta}_1$ = 0.08, 95% CI [-0.04, 0.20], *p* = .172, moderate BF_{null} = 3.99, and anecdotal evidence (1 < BF < 3) (8) that leaders with higher levels of ambition are rated as *no more effective* overall in their leadership role by their direct reports, $\hat{\beta}_1$ = 0.10, 95% CI [-0.02, 0.22], *p* = .091, anecdotal BF_{null} = 2.52.

In the second wave of data collection, we find moderate evidence that leaders with higher levels of ambition rate themselves as *more effective* overall in their leadership role, $\hat{\beta}_1$ = 0.12, 95% CI [0.04, 0.19], *p* = .003, moderate BF_{alt} = 5.99. However, we find strong evidence that leaders with higher levels of ambition are rated as *no more effective* overall in their leadership role by relevant third-party actors, $\hat{\beta}_1$ = 0.01, 95% CI [-0.04, 0.07], *p* = .680, strong BF_{null} = 20.39, or by their managers, $\hat{\beta}_1$ = 0.00, 95% CI [-0.08, 0.08], *p* = .947, strong BF_{null} = 14.10, and moderate evidence that leaders with higher levels of ambition are rated as *no more effective* overall in their leadership role by their peers, $\hat{\beta}_1$ = -0.04, 95% CI [-0.11, 0.03], *p* = .275, moderate BF_{null} = 9.28, or their direct reports, $\hat{\beta}_1$ = 0.06, 95% CI [-0.02, 0.13], *p* = .149, moderate BF_{null} = 5.92.

Exploratory Analysis Controlling for Extraversion

To further ascertain whether the observed null relationship between ambition and third-party ratings of effectiveness stems from ambition itself rather than other factors related to personality, we ran additional, exploratory analyses in which we control for extraversion. That is, we test whether the null relationship between ambition and third-party ratings of effectiveness holds when controlling for leader extraversion.

Extraversion was measured in the second wave of data collection, using the 6-item BFI-2-Short Scale (9). Participants were asked to indicate (on a 5-point scale ranging from "Disagree strongly" to "Agree strongly") the degree to which they are someone who "Tends to be quiet" (R), "Is dominant, acts as a leader", "Is full of energy", "Is outgoing, sociable", "Prefers to have others take charge" (R), and "Is less active than other people." We averaged the items to create a single measure of leader extraversion (coefficient alpha $\hat{\alpha}$ = 0.68, categorical omega $\widehat{\omega_C}$ = 0.72) (10).

When controlling for leader extraversion, we again find moderate to strong evidence *against* a relationship between ambition and third-party ratings of effectiveness. Using our main measure of ambition, we find strong evidence (10 < BF < 30) (8) that leaders with higher levels of ambition are rated as *no more effective* overall in their leadership role by all third-party actors, $\hat{\beta}_1$ = 0.01, 95% CI [-0.05, 0.06], *p* = .845, strong BF_{null} = 13.45, and moderate evidence (3 < BF < 10) (8) that leaders with higher levels of ambition are rated as *no more effective* overall in their leadership role by their managers, $\hat{\beta}_1$ = 0.00, 95% CI [-0.08, 0.08], *p* = .982, moderate BF_{null} = 8.72, their direct reports, $\hat{\beta}_1$ = 0.05, 95% CI [-0.03, 0.13], *p* = .186, moderate BF_{null} = 4.40, or their peers, $\hat{\beta}_1$ = -0.05, 95% CI [-0.12, 0.03], *p* = .219, moderate BF_{null} = 4.97.

We run the same exploratory analysis for drive to achieve (our additional measure of ambition obtained in the second wave of data collection). When controlling for leader extraversion, we again find moderate to strong evidence *against* a relationship between drive to achieve and third-party ratings of effectiveness. We find strong evidence (10 < BF < 30) (8) that leaders with higher levels of drive to achieve are rated as *no more effective* overall in their leadership role by their peers, $\hat{\beta}_1$ = 0.00, 95% CI [-0.10, 0.11], *p* = .944, strong BF_{null} = 10.01, and moderate evidence (3 < BF < 10) (8) that leaders with higher levels of drive to achieve are rated as *no more effective* overall in their leadership role by all third-party actors, $\hat{\beta}_1$ = 0.06, 95% CI [-0.02, 0.14], *p* = .133, moderate BF_{null} = 4.54, their managers, $\hat{\beta}_1$ = 0.08, 95% CI [-0.03, 0.19], *p* = .158, moderate BF_{null} = 3.32, and their direct reports, $\hat{\beta}_1$ = 0.07, 95% CI [-0.04, 0.17], *p* = .232,

moderate BF_{null} = 4.98.

Exploratory Analysis of Gender Moderation

Because ambition is a highly agentic quality and agency is closely associated with gender, we explore whether the relationship between ambition and leadership evaluations is moderated by gender. We ran mixed effects models predicting effectiveness ratings from ambition, gender, an interaction effect (β_3) between ambition and gender, and random intercepts for leaders and survey items.

We find moderate evidence (3 < BF < 10) that the relationship between ambition and effectiveness is *not moderated by gender* for self-evaluations, $\hat{\beta}_3$ = 0.02, 95% CI [-0.11, 0.14], *p* = .790, moderate BF_{null} = 9.33, all third-party evaluations, $\hat{\beta}_3$ = -0.05, 95% CI [-0.14, 0.05], *p* = .337, moderate BF_{null} = 9.12, manager evaluations, $\hat{\beta}_3$ = 0.03, 95% CI [-0.10, 0.17], *p* = .633, moderate BF_{null} = 7.56, direct report evaluations, $\hat{\beta}_3$ = -0.07, 95% CI [-0.21, 0.07], *p* = .346, moderate BF_{null} = 6.50, or peer evaluations, $\hat{\beta}_3$ = -0.03, 95% CI [-0.15, 0.09], *p* = .581, moderate BF_{null} = 9.15.

This result is in line with the work of Ma and colleagues (11), who argue that agency is comprised of six factors (competent agency, ambitious agency, dominant agency, diligent agency, independent agency, and self-assured agency) that differentially impact evaluations of female leaders. This work suggests that female leaders who display competent agency, diligent agency, and independent agency elicit an agentic *advantage* (i.e., they are evaluated more *favorably* than men), while women who display dominant agency elicit an agentic *disadvantage* (i.e., they are evaluated more *negatively* than men). For ambitious (and self-assured) agency, however, Ma and colleagues (11) do *not* find evidence of an agentic advantage or disadvantage, suggesting—in line with our findings—that gender does not moderate the relationship between ambition and leadership evaluations.

Exploratory Analysis by Leadership Competency: Motivation to Lead

In addition to our main, preregistered analyses, we ran an exploratory analysis of the ten leadership competencies. Specifically, we assess the degree of self-other discrepancy that appears for each competency. Figure S3 displays the relationship between *motivation to lead* (our preregistered, alternative measure of leader ambition) and evaluations of effectiveness in the leader role for each separate competency.

First, while we find extreme evidence (BF > 100) (8) that leaders with greater motivation to lead rate themselves as demonstrating greater accountability for results (e.g., taking projects to completion and accepting responsibility for mistakes), $\hat{\beta}_1$ = 0.27, 95% CI [0.16, 0.38], *p* < .001, extreme BF_{alt} = 2,473.87, we find moderate to strong evidence that relevant third-party actors, managers, peers, and direct reports *do not* observe greater accountability for results in leaders with greater motivation to lead (5.86 < BF_{null} < 11.64).

Second, while we find extreme evidence that leaders with greater motivation to lead rate themselves as demonstrating greater ability to present and communicate ideas (e.g., presenting complex material in a way that is clear and engaging and reinforcing messages with specific take-aways or action items), $\hat{\beta}_1$ = 0.33, 95% CI [0.19, 0.47], *p* < .001, extreme BF_{alt} = 1,248.77, we find anecdotal to strong evidence that relevant third-party actors, managers, and peers *do not* observe greater ability to present and communicate ideas in leaders with higher levels of ambition (2.80 < BF_{null} < 10.30). We do find, however, anecdotal evidence that direct reports observe greater ability to present and communicate ideas in leaders with greater motivation to lead, $\hat{\beta}_1$ = 0.16, 95% CI [0.02, 0.30], *p* = .029, anecdotal BF_{alt} = 1.10.

Third, while we find extreme evidence that leaders with greater motivation to lead rate themselves as demonstrating greater growth orientation (e.g., soliciting developmental feedback to improve performance and asking for opportunities to further career development), $\hat{\beta}_1$ = 0.28, 95% CI [0.14, 0.41], *p* < .001, extreme BF_{alt} = 237.47, we find anecdotal to strong evidence that relevant third-party actors, managers, peers, and direct reports *do not* observe greater growth orientation in leaders with greater motivation to lead (2.61 < BF_{null} < 11.91).

Fourth, while we find very strong evidence (30 < BF < 100) (8) that leaders with greater motivation to lead rate themselves as demonstrating greater ability to motivate others (e.g., understanding what motivates each person they work with and contributing to a strong sense of team identity), $\hat{\beta}_1$ = 0.23, 95% CI [0.10, 0.35], *p* = .001, very strong BF_{alt} = 32.61, we find moderate to strong evidence that relevant third-party actors, managers, peers, and direct reports *do not* observe greater ability to motivate others in leaders with greater motivation to lead (5.66 < BF_{null} < 10.27).

Fifth, while we find moderate evidence that leaders with greater motivation to lead rate themselves as demonstrating greater ability to exert influence and persuasion (e.g., building support for their initiatives and negotiating skillfully while maintaining good relationships), $\hat{\beta}_1$ = 0.22, 95% CI [0.07, 0.38], *p* = .005, moderate BF_{null} = 6.60, we find moderate evidence that relevant third-party actors, managers, peers, and direct reports *do not* observe greater ability to exert influence and persuasion in leaders with greater motivation to lead (5.41 < BF_{null} < 9.76).

Sixth, while we find moderate evidence that leaders with greater motivation to lead rate themselves as demonstrating greater ability to manage collaborative work (e.g., following up on incomplete tasks and getting buy-in for processes that involve multiple people), $\hat{\beta}_1$ = 0.20, 95% CI [0.06, 0.34], *p* = .007, moderate BF_{alt} = 4.21, we find moderate to strong evidence that relevant third-party actors, managers, peers, and direct reports *do not* observe greater ability to manage collaborative work in leaders with greater motivation to lead (7.97 < BF_{null} < 15.79).

Seventh, while we find anecdotal evidence that leaders with greater motivation to lead rate themselves as demonstrating greater ability to coach and develop people (e.g., investing time and energy to coach others and promising development opportunities for younger leaders), $\hat{\beta}_1$ = 0.19, 95% CI [0.04, 0.35], *p* = .017, anecdotal BF_{alt} = 2.41, we find moderate to strong evidence that relevant third-party actors, managers, peers, and direct reports *do not* observe greater ability to coach and develop people in leaders with greater motivation to lead (5.88 < BF_{null} < 12.63).

Eighth, while we find anecdotal evidence that leaders with greater motivation to lead rate themselves as demonstrating greater ability to self-manage (e.g., maintaining a positive attitude and recovering quickly from setbacks), $\hat{\beta}_1$ = 0.15, 95% CI [0.03, 0.27], *p* = .013, anecdotal BF_{alt} = 2.12, we find moderate to strong evidence that relevant third-party actors, managers, peers, and direct reports *do not* observe greater ability to self-manage in leaders with greater motivation to lead (8.83 < BF_{null} < 13.57).

For two of the ten leadership competencies, we find anecdotal to moderate evidence against a relationship between motivation to lead and self-evaluations of effectiveness in the leadership role. Leaders with greater motivation to lead *do not* rate themselves as demonstrating significantly greater decision-making ability, $\hat{\beta}_1$ = 0.09, 95% CI [-0.02, 0.19], *p* = .120, anecdotal BF_{null} = 2.85, or conflict resolution skills, $\hat{\beta}_1$ = 0.02, 95% CI [-0.14, 0.18], *p* = .805, moderate BF_{null} = 6.67. Similarly, relevant third-party actors, managers, peers, and direct reports *do not* observe greater decision-making ability (6.27 < BF_{null} < 13.33) or conflict resolution skills (1.95 < BF_{null} < 6.48) in leaders with greater motivation to lead.

Exploratory Analysis by Leadership Competency: Drive to Achieve

Figure S4 displays the relationship between drive to achieve and evaluations of effectiveness in the leader role by each leadership competency. First, while we find extreme evidence that leaders with higher drive to achieve rate themselves as demonstrating greater accountability for results, $\hat{\beta}_1$ = 0.31, 95% CI [0.19, 0.43], *p* < .001, extreme BF_{alt} = 6,901.49, we find anecdotal to moderate evidence that managers, peers, and direct reports *do not* observe greater accountability for results in leaders with higher drive to achieve (1.72 < BF_{null} < 6.48). We do find, however, anecdotal evidence of a relationship between drive to achieve and overall third-party evaluations of accountability for results, $\hat{\beta}_1$ = 0.11, 95% CI [0.03, 0.20], *p* = .009, anecdotal BF_{alt} = 1.56.

Second, while we find extreme evidence that leaders with higher drive to achieve rate themselves as demonstrating greater ability to motivate others, $\hat{\beta}_1$ = 0.34, 95% CI [0.18, 0.50], *p* < .001, extreme BF_{alt} =

506.74, we find anecdotal to moderate evidence that managers, peers, and direct reports *do not* observe greater ability to motivate others in leaders with higher drive to achieve (1.80 < BF$_{null}$ < 7.97). We do find, however, anecdotal evidence of a relationship between drive to achieve and overall third-party evaluations of ability to motivate others, $\hat{\beta}_1$ = 0.13, 95% CI [0.03, 0.23], *p* = .015, anecdotal BF$_{alt}$ = 1.20.

Third, while we find moderate evidence that leaders with higher drive to achieve rate themselves as demonstrating greater ability to manage collaborative work, $\hat{\beta}_1$ = 0.23, 95% CI [0.07, 0.38], *p* = .004, moderate BF$_{alt}$ = 5.58, we find moderate to strong evidence that relevant third-party actors, managers, peers, and direct reports *do not* observe greater ability to manage collaborative work in leaders with higher drive to achieve (3.96 < BF$_{null}$ < 13.82).

Fourth, while we find moderate evidence that leaders with higher drive to achieve rate themselves as demonstrating greater growth orientation, $\hat{\beta}_1$ = 0.21, 95% CI [0.07, 0.36], *p* = .004, moderate BF$_{alt}$ = 4.82, we find anecdotal to moderate evidence that managers, peers, and direct reports *do not* observe greater growth orientation in leaders with higher drive to achieve (1.51 < BF$_{null}$ < 5.30). We do find, however, anecdotal evidence of a relationship between drive to achieve and overall third-party evaluations of growth orientation, $\hat{\beta}_1$ = 0.11, 95% CI [0.03, 0.19], *p* = .009, anecdotal BF$_{alt}$ = 1.42.

Fifth, while we find moderate evidence that leaders with higher drive to achieve rate themselves as exerting greater influence and persuasion, $\hat{\beta}_1$ = 0.23, 95% CI [0.07, 0.40], *p* = .006, moderate BF$_{alt}$ = 4.29, we find moderate evidence that peers and direct reports *do not* observe greater ability to exert influence and persuasion in leaders with higher drive to achieve (5.12 < BF$_{null}$ < 8.44). We do find, however, anecdotal evidence that third-party raters and managers observe greater ability to exert influence and persuasion in leaders with higher drive to achieve (1.08 < BF$_{alt}$ < 1.10).

Sixth, while we find anecdotal evidence that leaders with higher drive to achieve rate themselves as demonstrating greater ability to coach and develop people, $\hat{\beta}_1$ = 0.23, 95% CI [0.05, 0.41], *p* = .011, anecdotal BF$_{alt}$ = 2.89, we find anecdotal to moderate evidence that relevant third-party actors, managers, peers, and direct reports *do not* observe greater ability to coach and develop people in leaders with higher drive to achieve (1.91 < BF$_{null}$ < 9.77).

Seventh, while we find anecdotal evidence that leaders with higher drive to achieve rate themselves as demonstrating greater decision-making ability, $\hat{\beta}_1$ = 0.15, 95% CI [0.02, 0.27], *p* = .022, anecdotal BF$_{alt}$ = 1.19, we find moderate to strong evidence that relevant third-party actors, managers, peers, and direct reports *do not* observe greater decision-making ability in leaders with higher drive to achieve (9.69 < BF$_{null}$ < 17.19).

For three of the ten leadership competencies, we find anecdotal to moderate evidence against a relationship between drive to achieve and self-evaluations of effectiveness in the leadership role. Leaders with greater drive to achieve *do not* rate themselves as demonstrating significantly greater communication skills (anecdotal BF$_{null}$ = 1.21), ability to self-manage (moderate BF$_{null}$ = 3.93), or conflict resolution skills (moderate BF$_{null}$ = 8.42). Similarly, relevant third-party actors, managers, peers, and direct reports *do not* observe greater communication skills (7.63 < BF$_{null}$ < 11.77), ability to self-manage (5.52 < BF$_{null}$ < 17.13), or conflict resolution skills (5.58 < BF$_{null}$ < 13.16) in leaders with greater drive to achieve (note that peers observe significantly *lower* conflict resolution skills in leaders with greater drive to achieve, $\hat{\beta}_1$ = -0.16, 95% CI [-0.29, -0.03], *p* = .019, anecdotal BF$_{alt}$ = 1.19).

Exploratory Response Surface Analysis

As an additional exploratory analysis, we ran a Response Surface Analysis to examine the simultaneous relationship between ambition, self-perceptions, and third-party perceptions. This strategy allows us to simultaneously test whether ambition accounts *only* for positive self-views and *not* for positive third-party perceptions of leader effectiveness.

We use Response Surface Analysis to examine how ambition relates to both self-views and third-party views of leader effectiveness. We follow the information-theoretic approach described in detail by

Humberg and colleagues (12). First, we estimate all plausible polynomial models relating ambition to self-views of leader effectiveness, third-party views of leader effectiveness, and their interplay. Second, we reduce the model set by removing redundant models (i.e., in the case of two nested models with log-likelihoods that differ by less than 1, we exclude the model with more free parameters). Next, we identify the best fitting polynomial model—defined as the model with the smallest second-order Akaike Information Criterion (AICc) or, equivalently, the greatest Akaike weight—and plot the estimated regression surface. Crucially, this approach allows us to test whether ambition increases the positivity of self-perceptions regardless of the extent to which this self-perception is corroborated by relevant actors, including peers, subordinates, and managers.

Our hypothesis that ambition is related to favorable self-perceptions, but not to favorable third-party perceptions, is represented by the Uncorroborated Positive Self-Views model (referred to as the "Beneficial Positivity of Self-View Only Model" in Humberg et al.) (12: p. 841). This model imposes two constraints on the regression surface: (a) holding third-party perceptions constant, ambition is posited to have a positive relationship with self-views of leader effectiveness (i.e., $\beta_1 > 0$); and (b) holding self-views constant, ambition is posited to have no relationship with third-party perceptions of leader effectiveness (i.e., $\beta_2 = 0$). Thus, we infer additional, exploratory support for our hypotheses if a Response Surface Analysis indicates that the Uncorroborated Positive Self-Views model has the best fit with the data out of all plausible polynomial models relating ambition to self-views of leader effectiveness, third-party views of leader effectiveness, and their interplay.

To simultaneously test whether ambition accounts *only* for positive self-views and *not* for positive third-party perceptions of leader effectiveness, we ran a separate Response Surface Analysis for each of our three measures of ambition. Figure S5 plots the estimated regression surfaces for ambition, motivation to lead, and drive to achieve, respectively. As demonstrated by the estimated regression surfaces, the Uncorroborated Positive Self-Views model provides the best explanation for the data in all three cases, with Akaike weights w = 0.91, 0.94, and 0.97. When controlling for third-party perceptions, self-views of leader effectiveness are positively associated with ambition, $\hat{\beta}_1 = 0.28$, 95% CI [0.14, 0.42], $z = 3.93$, $p < .001$, motivation to lead, $\hat{\beta}_1 = 0.48$, 95% CI [0.27, 0.68], $z = 4.59$, $p < .001$, and drive to achieve, $\hat{\beta}_1 = 0.22$, 95% CI [0.11, 0.34], $z = 3.77$, $p < .001$. In line with the best fitting model and our hypotheses, third party perceptions have no relationship with ambition when controlling for self-views (i.e., $\beta_2 = 0$).

Pilot Studies

Pilot 1: Experimental Study of Lay Perceptions

Ambition shares conceptual overlap with several qualities that are considered prototypical of leaders, including dedication (e.g., motivation, hard work, determination, and goal-orientedness) (13–16), conscientiousness (17, 18), achievement striving (17, 19), and agency (11). We ran an experiment to test whether lay theories about effective leadership indeed favor ambitious individuals.

We recruited a sample of 151 online participants. We randomly assigned these participants to read a vignette that described either an individual with high ambition or an individual with low ambition. We modeled the experimental manipulation after the ambition scale used throughout the paper. In the low ambition condition, participants read "John is not very ambitious when it comes to his career goals. He does not want to attain a high-status position in his career and is not highly motivated to get promoted quickly or often." In the high ambition condition, participants read "John has strong ambition when it comes to his career goals. He wants to attain a high-status position in his career and is highly motivated to get promoted quickly and often."

Next, participants rated the target individual's leadership potential using three items on a scale from 1 (Strongly Disagree) to 7 (Strongly Agree): "This person would make a capable leader", "This person has the qualities of a highly effective leader", and "This person likely has excellent leadership skills" (20) (coefficient alpha $\hat{\alpha} = 0.97$, categorical omega $\widehat{\omega_C} = 0.97$) (10). As shown in Figure S6, participants who read a vignette about a more ambitious individual rated that person as having greater leadership potential (M = 4.89, SD = 1.02, n = 74) than did participants who read about a less ambitious individual (M = 2.26, SD = 1.22, n = 77), $\hat{\beta} = 2.83$, 95% CI [2.27, 2.99], $t(149) = 14.33$, $r = .76$, 95% CI [.68, .82], Cohen's d =

2.34, $p < .001$. In other words, at first blush, lay individuals *assume* that ambitious individuals will be more effective as leaders.

Pilot 2: Time-Lagged Study of Leader Emergence

To assess the effects of leadership ambition on self-views and leader emergence in a naturally occurring setting, we ran an archival study using time-lagged survey data obtained from the Higher Education Research Institute (HERI) at UCLA. The goal of this pilot study was twofold. First, we aimed to assess the cross-sectional association between ambition and self-rated leadership ability (H1). Second, we aimed to assess the time-lagged effect of ambition as a college freshman on leader emergence as a college senior (Supplemental Hypothesis). The study design, data collection procedure, hypotheses, statistical models, and code were preregistered at the OSF (https://osf.io/btynm?view_only=af7f64e52c37443ca62103b44c78cec4).

Sample and Procedure

We obtained the yearly Freshman Survey (available from 1966 to 2010) and the yearly College Senior Survey (available from 1994 to 2008) from the HERI Data Archives. The Freshman Survey contains data for 14,235,487 college freshmen at 1,840 institutions in the United States (52.9% female; 79.3% White, 7.9% Black, 4.5% Asian, 3.1% Hispanic, 0.3% Native American, 4.8% Other Race/Ethnicity). The College Senior Survey contains 527,159 college seniors at 722 institutions in the United States (62.5% female; 78.0% White, 5.4% Black, 4.9% Asian, 4.6% Hispanic, 6.8% Other Race/Ethnicity). Responses from participants in the College Senior Survey are matched to their responses to the Freshman Survey, allowing us to assess the effect of psychological variables (e.g., ambition) on behaviors (e.g., leadership emergence) over time. We refer to the Freshman Survey as T1 (Time 1) and the Senior Survey as T2 (Time 2).

Measures

We report all measures collected for Pilot S2. All measures, including the exact time points at which they were assessed, were preregistered at the OSF Registries (https://osf.io/btynm?view_only=af7f64e52c37443ca62103b44c78cec4).

Ambition. We collected two measures of ambition: a main measure to serve as the independent variable (self-rated drive to achieve) and an additional measure to serve as a robustness check (importance of becoming a community leader). For the main measure of ambition, respondents rated themselves on drive to achieve, compared to the average person their age, using a 5-point scale (Highest 10%, Above Average, Average, Below Average, Lowest 10%). For the additional measure of ambition, respondents indicated the importance to themselves personally of becoming a community leader, using a 4-point scale (Essential, Very Important, Somewhat Important, Not Important). Both measures were obtained at T1 and T2.

Leadership ability. Respondents rated themselves on leadership ability, compared to the average person their age, on a 5-point scale (Highest 10%, Above Average, Average, Below Average, Lowest 10%). This measure was obtained at T1 and T2.

Leader emergence. We used two measures of leadership behavior: participation in leadership training and participation in student government. Both behaviors were assessed only in the College Senior Survey (T2). Respondents indicated whether, since entering college, they participated in leadership training (Yes/No) or participated in student government (Yes/No). We did not combine these two items into a single index; rather, the two behaviors were treated as separate indicators of leader emergence.

Data Analysis

All hypotheses, statistical models, code, and inference criteria were preregistered at the OSF (https://osf.io/btynm?view_only=af7f64e52c37443ca62103b44c78cec4). For H1, we preregistered a main test and three robustness checks to assess whether ambition is positively associated with self-rated

leadership ability. For the main test, we assess the correlation between drive to achieve (T1) and self-rated leadership ability (T1). To assess whether the same relationship holds for a different measure of ambition, we include a robustness check, instead assessing the correlation between personal importance of becoming a community leader (T1) and self-rated leadership ability (T1). To assess whether the same relationship holds at alternative time points, we include two robustness checks. First, we assess whether the relationship observed among freshmen also holds for seniors (i.e., we assess the correlation between drive to achieve at T2 and self-rated leadership ability at T2). Second, we assess whether the observed relationship holds over time (i.e., we assess the correlation between drive to achieve at T1 and self-rated leadership ability at T2). For the main test of H1, we reject the null if (a) the *p*-value of the Pearson correlation coefficient is less than 0.05 and (b) the correlation coefficient is in the predicted direction (i.e., positive).

For the Supplemental Hypothesis, we preregistered two main tests and two robustness checks. For the main tests, we ran two separate logistic regression models to predict leadership emergence (T2) from drive to achieve (T1). Each of the two models uses a different indicator of leader emergence: for the first model, we predict participation in student government (T2) from drive to achieve (T1); for the second model, we predict participation in leadership training (T2) from drive to achieve (T1). To assess whether the same relationship holds for a different measure of ambition, we include two robustness checks, this time predicting each of the two indicators of leader emergence (T2) from self-rated importance of becoming a community leader (T1). Because we ran two separate logistic regression models, we use the Bonferroni method to correct for multiple comparisons. We reject the null and retain the alternative hypothesis if for both regression models that serve as the main test of the Supplemental Hypothesis (a) the *p*-value for the slope is less than 0.025 and (b) the slope is in the predicted direction (i.e., positive).

Results

Table S3 reports basic descriptive statistics and correlations. H1 predicts a positive association between ambition and self-rated leadership ability. In Figure S7, we plot the relationship between drive to achieve and average self-ratings of leadership ability for our main test and two robustness checks. As can be inferred from Figure S7 (panel one), the correlation between drive to achieve (T1) and self-rated leadership ability (T1) is positive, $\hat{\rho}$ = 0.397, 95% CI [0.396, 0.397], $t(9{,}648{,}265) = 1{,}343.02$, $p < .001$.

As a robustness check, we evaluate whether the positive relationship between ambition and self-rated leadership ability holds at alternative timepoints. Further strengthening the evidence in favor of H1, the relationship found among college freshman also holds over time (Figure S7 panel two): students with higher levels of ambition as freshmen (T1) rate themselves higher on leadership ability as seniors (T2), $\hat{\rho}$ = 0.26, 95% CI [0.256, 0.263], $t(256{,}047) = 136.08$, $p < .001$. This relationship also holds for college seniors (Figure S7 panel three): drive to achieve (T2) and self-rated leadership ability (T2) are positively correlated, $\hat{\rho}$ = 0.42, 95% CI [0.418, 0.422], $t(487{,}598) = 323.16$, $p < .001$. As a final robustness check, we assessed the correlation between personal importance of becoming a community leader (T1) and self-rated leadership ability (T1). The positive correlation also holds for this alternative measure of ambition, $\hat{\rho}$ = 0.391, 95% CI [0.391, 0.392], $t(6{,}105{,}415) = 1{,}050.87$, $p < .001$.

The Supplemental Hypothesis predicts a higher probability of leader emergence for college seniors who have higher levels of ambition as freshmen. In Figure S8, we plot the average participation rates in student government and leadership training, respectively, as a function of T1 drive to achieve. As can be inferred from Figure S8 (panels one and two, respectively), we find a positive effect of drive to achieve (T1) on the log odds of participation in student government (T2), $\hat{\beta}$ = 0.32, 95% CI [0.30, 0.33], $z = 40.26$, $p < .001$, and on the log odds of participation in leadership training (T2), $\hat{\beta}$ = 0.33, 95% CI [0.32, 0.35], $z = 50.78$, $p < .001$.

Translating the log odds to the probabilities of leader emergence for students who rated their drive to achieve (compared to the average person their age) as being in the lowest 10% vs. the highest 10%, we find that students with the lowest level of ambition as freshmen have a 5% chance of participating in student government over time, while students with the highest level of ambition have a 17% chance of participating in student government. Similarly, students with the lowest level of ambition as freshmen have

a 10% chance of participating in leadership training over time, compared to a 30% chance for students with the highest level of ambition.

As a robustness check, we evaluated whether the positive relationship between ambition (T1) and leader emergence (T2) holds for an alternative measure of ambition. In Figure S9, we plot the average participation rates in student government and leadership training (panels one and two, respectively) as a function of T1 importance of becoming a community leader. Strengthening the evidence in favor of the Supplemental Hypothesis, we find a positive effect of the rated importance of becoming a community leader (T1) on participation in student government (T2), $\hat{\beta} = 0.52$, 95% CI [0.51, 0.53], $z = 74.02$, $p < .001$, and on participation in leadership training (T2), $\hat{\beta} = 0.44$, 95% CI [0.43, 0.45], $z = 78.76$, $p < .001$.

Translating the log odds to the probabilities of leader emergence for freshmen who indicate that becoming a community leader is personally unimportant vs. essential to them, students with the lowest level of ambition as freshmen have a 7% chance of participating in student government over time, compared to a 26% chance for students with the highest level of ambition. Similarly, students with the lowest level of ambition (measured by their rated personal importance of becoming a community leader) as freshmen have a 16% chance of participating in leadership training over time, compared to a 42% chance for students with the highest level of ambition.

Taken together, the results from Pilot S2 present strong observational evidence of a cross-sectional relationship between ambition and self-rated leadership ability (H1) as well as a positive time-lagged relationship between ambition and leader emergence (Supplemental Hypothesis).

There are two potential explanations for the found relationship between ambition and leader emergence. Leader emergence can stem either from opt-in processes (i.e., ambitious individuals may be more likely to *self-select* into leadership roles) or from the active selection of individuals who signal effectiveness (i.e., ambitious individuals may be more likely to be selected for leadership roles because third parties view ambition as a positive *signal*). An important limitation of Pilot Study 2 is that our data cannot speak to the process underlying the time-lagged effect of ambition on emergence. An interesting question for future research to explore is whether lay beliefs about the relationship between leadership ambition and leadership aptitude contribute to leader emergence (i.e., whether the link between ambition and emergence stems from *signaling* processes).

Exploratory Test of Mechanism: Above Average Effects

In addition to directly testing whether ambition is associated with self-views of leadership ability and rates of leader emergence, we can use the pilot data to test one of the proposed mechanisms in the main paper. That is, are ambitious individuals more prone to the “Above-Average” effect? In freshman and senior year of college, respondents were asked to indicate their leadership ability compared to the average person their age: Lowest 10%, Below Average, Average, Above Average, or Highest 10%. We coded the “Above Average” and “Highest 10%” responses as indicating an “Above-Average Belief.”

Next, we ran logistic regression models to test whether ambition (i.e., drive to achieve) increases the proportion of “above-average” leadership ability beliefs. We find a positive relationship between drive to achieve (T1) and the log odds of holding above-average beliefs about leadership ability in freshman year of college (T1), $\hat{\beta} = 0.92$, 95% CI [0.92, 0.92], $z = 999.81$, $p < .001$, and in senior year of college (T2), $\hat{\beta} = 0.61$, 95% CI [0.60, 0.62], $z = 110.19$, $p < .001$.

Translating the log odds to the proportion of above-average beliefs, we find that the proportion of above-average beliefs *in freshmen year* increases from 8% (for students with the lowest drive to achieve at T1) to 78% (for students with the highest drive to achieve at T1), and the proportion of above-average beliefs *in senior year* increases from 22% (for students with the lowest drive to achieve at T1) to 77% (for students with the highest drive to achieve at T1). In other words, over three-quarters of highly ambitious freshmen believe that they are above average in leadership ability (both in freshman and senior year of college).

For ambitious seniors, we find even stronger above-average effects. We find a positive relationship between drive to achieve (T2) and the log odds of holding above-average beliefs about leadership ability in senior year of college, $\hat{\beta}$ = 0.97, 95% CI [0.96, 0.98], z = 230.39, p < .001.

Translating the log odds to the proportion of above-average beliefs, we find that the proportion of above-average beliefs *in senior year* increases from 9% (for students with the lowest drive to achieve at T2) to 83% (for students with the highest drive to achieve at T2). In other words, over 80% of highly ambitious seniors believe that they are above average in leadership ability.

We ran the same models for our second measure of ambition: rated personal importance of becoming a community leader. We find a positive relationship between rated importance of becoming a community leader (T1) and the log odds of holding above-average beliefs about leadership ability in freshman year of college (T1), $\hat{\beta}$ = 0.83, 95% CI [0.82, 0.83], z = 779.83, p < .001, and in senior year of college (T2), $\hat{\beta}$ = 0.64, 95% CI [0.63, 0.65], z = 115.23, p < .001.

Translating the log odds to the proportion of above-average beliefs, we find that the proportion of above-average beliefs *in freshmen year* increases from 37% (for students with the lowest rated importance at T1) to 87% (for students with the highest rated importance at T1), and the proportion of above-average beliefs *in senior year* increases from 47% (for students with the lowest rated importance at T1) to 86% (for students with the highest rated importance at T1). In other words, around 85% of highly ambitious freshmen—in terms of wanting to become a community leader—believe that they are above average in leadership ability (both in freshman and senior year of college).

For seniors, we find similarly strong evidence of above-average beliefs. We find a positive relationship between rated importance of becoming a community leader (T2) and the log odds of holding above-average beliefs about leadership ability in senior year of college, $\hat{\beta}$ = 0.81, 95% CI [0.81, 0.82], z = 205.20, p < .001. Translating the log odds to the proportion of above-average beliefs, we find that the proportion of above-average beliefs in senior year increases from 41% (for students with the lowest drive to achieve at T2) to 89% (for students with the highest drive to achieve at T2). In other words, close to 90% of highly ambitious seniors—in terms of wanting to become a community leader—believe that they are above average in leadership ability.

Overall, the results of Pilot 2 strongly suggest that ambitious individuals are especially prone to above-average effects in the domain of leadership ability ratings.

Supplemental Studies

Supplemental Study 1: MBA Leadership Challenge

Supplemental Study 1 (N = 829 ratings of 181 MBA students) aimed to examine the possible relationship between leadership ambition and third-party ratings of leadership effectiveness with an ecologically valid, naturally occurring leadership simulation that involved expert evaluators. Participants were first-year MBA students enrolled in a West Coast business school in the United States. As part of the curriculum, all students took a leadership skills course that culminated in a one-day leadership skills competition. A month before the competition, each student received an invitation to participate in our study (an email containing a link to an online survey). We informed students that participation was voluntary and would not affect their course grade. 192 out of all 418 first-year MBA students agreed to participate in the pre-competition survey (M_{age} = 27.45, SD_{age} = 2.10, 46% female, 46% response rate).

At the start of the competition, all students were assigned to pairs and given one hour to review their simulation materials and prepare for their presentation later that day. Each pair was assigned one of three different leadership simulations that highlight a unique “leadership challenge.” To accommodate all 209 student pairs, 36 separate classroom spaces were utilized, with each hosting three “rounds” of leadership simulations. Each round consisted of two 20-minute presentations—two pairs of students presented in each round. After each round, students rotated to new rooms, while the judges remained in the same room. Thus, the judges evaluated the same leadership simulation throughout the entire day. Each room

had five judges, on average, who evaluated the students' leadership skills based on their performance in the simulation.

A total of 187 judges participated in the event (43% female). Almost all judges were alumni of the MBA program who are, or have been, executives across a range of industries (a small number of faculty members also participated as judges). The judges held an average of 36 years post-MBA work experience and job titles such as VP, CEO, and Managing Director. After each presentation, judges anonymously rated each individual presenter on their leadership effectiveness. Due to missing ratings from several judges, the final sample used in our analysis included 181 of the 192 MBA students who completed our survey (M_{age} = 27.48, SD_{age} = 2.13, 46% female, 51% European American/White, 6% Black/African American, 11% Asian/Pacific Islander, 22% Hispanic/Latino, 6% Middle Eastern, 5% Other).

Simulation Descriptions

In one case, the two students play the role of a CEO and CTO for a space satellite start-up (Astromech) currently raising its Series B round. After failing to meet certain performance milestones, the two leaders are now meeting with their primary investors (SUN Ventures) who are reluctant to proceed with another round of funding. During the meeting, the leaders must "explain the problems Astromech faces, recommend solutions, respond to investor concerns regarding the setbacks, and persuade SUN Ventures to proceed with its Series B investment and thereby save the round."

In another case, the students play the role of a COO and CTO of a "bespoke server company" called Teknika. The two leaders have developed a new concept "ideally suited to optimize servers for mobile and cloud applications." The CEO has asked them to gather input from the management team on the options for taking the idea from concept to production: "through Teknika's own internal R&D, through a skunk works, or by spinning out a new company." The two leaders are pre-disposed to a spin-out, but the rest of the management team disagrees. Their challenge is to manage the discussion in a way that clarifies the best option—one that maximizes the use of the firm's human and financial resources.

In a third case, the two students play the roles of CEO and Global Manager for a major beer company called "Brakan Beer," which has an opportunity to expand its operations in a foreign country called "Grinla." Grinla has 350 million people, but limited beer production. "Its government has been, and remains, a corrupt regime." Thus, the market opportunity is tantalizing, but the political climate is highly uncertain. The two leaders must formulate a recommendation, meet with Brakan's board, and convince at least three-quarters of the board to support their recommendation.

Measures

In line with the other studies, we assessed ambition using a 7-item, self-report measure (adapted from 1, 2) (coefficient alpha $\hat{\alpha}$ = 0.79, categorical omega $\widehat{\omega_C}$ = 0.85) (10). In addition, we included a self-report measure of status motives adapted from Neel and colleagues (2) as a robustness check for ambition. Participants indicated their agreement with three items, ranging from 1 (Strongly Disagree) to 7 (Strongly Agree): "It's important to me that other people look up to me", "I want to be in a position of leadership", and "It's important to me that others respect my rank or position" (coefficient alpha $\hat{\alpha}$ = 0.62, categorical omega $\widehat{\omega_C}$ = 0.66) (10).

For our measure of effectiveness, each expert judge rated each presenter using two items, "This person has all the qualities that would make him or her an effective leader" and "This person would succeed as a leader in my company." Responses were provided on a 7-point scale ranging from 1 = Strongly Disagree to 7 = Strongly Agree (coefficient alpha $\hat{\alpha}$ = 0.90, categorical omega $\widehat{\omega_C}$ = 0.91) (10).

To further ascertain whether the observed relationship between ambition and third-party ratings of effectiveness stems from ambition itself rather than other factors related to personality, we also collected a ten-item measure of personality (the ten-item personality inventory "TIPI") (21). Responses were provided on a 7-point scale ranging from 1 = Disagree Strongly to 7 = Agree Strongly. For example, to measure extraversion, participants indicated whether they see themselves as "Extraverted, enthusiastic" and "Reserved, quiet" (R) (coefficient alpha $\hat{\alpha}$ = 0.83, categorical omega $\widehat{\omega_C}$ = 0.85). In addition to our main

measures of interest, we collected self-report measures of guilt and shame proneness (the GASP scale) (22), dominance and prestige (23), and rank preference, for unrelated research.

Results

Table S4 reports basic descriptive statistics and correlations. We first checked for non-response bias by comparing the ratings of leadership effectiveness for those individuals who completed our survey to the ratings of those individuals who did not. We do not observe evidence of non-response bias in our data: results revealed no significant difference in leadership effectiveness based on survey completion, $\hat{\beta}$ = 0.05, 95% CI [-0.13, 0.24], $t(392.45) = 0.55$, $p = .585$.

We predicted a lack of association between ambition and third-party ratings of leadership effectiveness. For first-year MBA students participating in a leadership skills competition (N = 829 ratings of 181 MBA students), the relationship between ambition and expert-rated effectiveness was negative and non-significant, $\hat{\beta}_1$ = -0.10, 95% CI [-0.28, 0.07], $p = .241$. We find moderate evidence (3 < BF < 10) (8) that leaders with higher levels of ambition are rated as *no more effective* overall in their leadership role by expert judges, moderate BF_{null} = 3.63.

Next, we assess whether this relationship holds when we control for the “Big Five” personality traits (openness, conscientiousness, extraversion, agreeableness, and neuroticism). When controlling for core personality traits, we find anecdotal evidence (1 < BF < 3) (8) that leaders with higher levels of ambition are rated as *no more effective* overall in their leadership role by expert judges, $\hat{\beta}_1$ = -0.13, 95% CI [-0.31, 0.05], $p = .156$, anecdotal BF_{null} = 1.32 (note that here the evidence for the null is anecdotal because, if anything, there appears to be a slight *negative* relationship between ambition and expert-rated effectiveness).

As a robustness check, we also assess the relationship between status motives and expert-rated effectiveness. We again find moderate evidence *against* a relationship between status motives and expert-rated effectiveness, $\hat{\beta}$ = 0.05, 95% CI [-0.10, 0.21], $p = .500$, moderate BF_{null} = 5.61. When we control for the “Big Five” personality traits, we again find moderate evidence *against* a relationship between status motives and expert-rated effectiveness, $\hat{\beta}$ = 0.00, 95% CI [-0.16, 0.16], $p = .993$, moderate BF_{null} = 3.09.

Validity Check

An alternative explanation for why the data favored the null hypothesis is that our leadership effectiveness measure lacked validity (i.e., our items failed to capture the construct the way we intended). To address this concern, we examined the link between extraversion and ratings of leadership effectiveness. Meta-analyses strongly suggest that extraversion is one of the most reliable predictors of leadership evaluations (7). Therefore, finding evidence that extraversion positively correlates with ratings of leadership effectiveness in our data would support the predictive validity of our dependent variable. As expected, we find a positive correlation between extraversion and expert-rated leadership effectiveness, $r = .19$, 95% CI [.04, .33], $t(179) = 2.57$, $p = .011$, moderate BF_{alt} = 7.91, which casts doubt on the possibility that the lack of a significant relationship between ambition and third-party ratings of leadership effectiveness is due to a lack of validity for our measure of effectiveness.

Exploratory Analysis of Gender Moderation

Because ambition is a highly agentic quality and agency is closely associated with gender, we explore whether the relationship between ambition and leadership evaluations is moderated by gender. We ran mixed effects models predicting effectiveness ratings from ambition, gender, an interaction effect (β_3) between ambition and gender, and random intercepts for leaders and survey items.

We find anecdotal evidence (1 < BF < 3) that the relationship between ambition and third-party ratings of leadership effectiveness is *not moderated by gender*, $\hat{\beta}_3$ = -0.14, 95% CI [-0.50, 0.21], $p = .423$, anecdotal BF_{null} = 2.87. For our second measure of ambition, we find moderate evidence (3 < BF < 10) that the relationship between status motives and third-party ratings of leadership effectiveness is *not moderated by*

gender, $\hat{\beta}_3$ = -0.05, 95% CI [-0.36, 0.26], *p* = .751, moderate BF_{null} = 3.62.

Conclusion

Results from Supplemental Study 1 suggest no reliable relationship between ambition and expert-rated leadership effectiveness. Using a naturally occurring, large-scale behavioral leadership simulation, which offers a combination of rich ecological validity and a highly controlled environment, we find that ambition is not associated with third-party ratings of leadership effectiveness. These results are supportive of our theorizing, but the main study (reported in the main paper) and Supplemental Study 1 do have an important limitation. Namely, the evidence pointing to no relationship between ambition and third-party ratings of leadership effectiveness could stem from the unique characteristics of our samples, given that MBA students and executives generally score high on ambition. This may represent a restricted range, leading us to claim no relationship between ambition and third-party ratings of effectiveness when, at a population level, such a relationship may in fact appear. While the absence of a relationship in this high-achieving sample is interesting in and of itself, our findings would be more generalizable if we found support for the null hypothesis using a sample that is more representative of the general population. Supplemental Study 2 was designed with this exact concern in mind.

Supplemental Study 2: Nationally Representative Sample with Randomly Selected Leaders

In Supplemental Study 2 (*N* = 406), we recruited a nationally representative sample (i.e., based on the United States Census) to participate in an hour-long leadership simulation that focused on a group decision-making task. Participants were randomly assigned to act as the leader (*n* = 101) or as one of three followers (*n* = 305). All sample sizes, hypotheses, study measures, and analysis plans were preregistered at AsPredicted.org: https://aspredicted.org/blind.php?x=9vj8ua. To build our sample, we partnered with Bovitz, Inc., a company that specializes in compiling nationally representative survey panels. We aimed to collect data from 100 groups (i.e., 400 participants), representative with respect to age, gender, race, and income.

The second author acted as the experimenter for all 19 study sessions. A research assistant blind to the study hypotheses randomly assigned participants to 4-person groups and to roles within each group. Participants learned they would work together to select a new CFO for a fictitious company called “PB Technologies” (adapted from the case, *PB Technologies*, from the Dispute Resolution Research Center). Participants further learned they had been assigned to one of four Senior VP roles (Operations, General Counsel, Marketing, Human Resources) and would read a role-specific handout that provided detailed information about three potential candidates. They had 12 minutes to review the information. After they finished, they would discuss the candidates in their groups and come to a group decision. They were instructed to close the page with the candidate information before going into their group discussion, but they could take notes on the material they read and discuss it with their groups.

After these instructions were given, participants read through their role handouts, and, once 12 minutes passed, indicated in the survey which candidate they preferred. At that point, participants in the role of Senior VP of Marketing learned from the instructions that they would be the leader during the group discussion. Specifically, they would be “responsible for guiding the group’s discussion and helping the group come to a final agreement.” The experimenter repeated this information out loud to all participants so that it was clear to everyone that the Senior VP of Marketing was the group leader. The experimenter then sent participants into their breakout rooms for 20 minutes. If anyone dropped out of the session before the group discussion began, remaining group members were dismissed because all four group members needed to be present to ensure the group had complete information about the candidates. After 20 minutes, the experimenter closed the breakout rooms. After returning to the main Zoom room, participants navigated to the survey, identified which candidate their group chose, and completed the remaining survey questions.

The final sample included 406 participants from 102 groups (53% female, M_{age} = 43.54, SD_{age} = 15.22). The sample size is not a multiple of 4 because the Qualtrics survey site malfunctioned for two participants. We include data from their group members who were able to complete the survey as they all participated in the group discussion. We obtained a diverse sample with respect to race, gender, age, income, and

geographic location (see Supplemental Online Materials for demographic data on our sample versus the most recent U.S. Census data). Participants also occupied a wide range of industries, with the top three most represented categories being “professional, scientific, or technical services” (7%), “educational services” (10%), and “health care or social assistance” (7%).

Measures

In line with the other studies, we assessed ambition using a 7-item, self-report measure (adapted from 1, 2) (coefficient alpha $\hat{\alpha}$ = 0.92, categorical omega $\widehat{\omega_C}$ = 0.95). In line with the main study, we also included “motivation to lead” as an additional measure of ambition. We used Chan and Drasgow’s (24) 9-item Affective-Identity subscale (coefficient alpha $\hat{\alpha}$ = 0.90, categorical omega $\widehat{\omega_C}$ = 0.93).

For our measure of other-rated effectiveness, team members completed a 5-item measure of leadership effectiveness adapted from Schaumberg and Flynn (20). Specifically, participants were asked, “Think about the group member who was assigned to be the leader in your group (the Senior VP of Marketing). Please answer the questions below, as they apply to the leader in your group.” The items included, “I would want this person to be my boss or supervisor at work”, “This person has clear leadership potential”, “Overall, this person was NOT an effective leader” (reverse-scored), “If I had the chance, I would definitely want to have this person as my leader again”, and “Looking ahead, I expect this person will experience great success as a leader” (coefficient alpha $\hat{\alpha}$ = 0.93, categorical omega $\widehat{\omega_C}$ = 0.94).

As a robustness check, we included “empowering leader behavior” as an additional measure of other-rated leader effectiveness, adapted from Arnold and colleagues (25). This 6-item measure used three items from the “coaching” subscale and three from the “participative decision-making” subscale that were relevant to the simulation task. Specifically, participants were asked how often the leader of their group, “Encouraged group members to express their ideas/suggestions”, “Listened to the group’s ideas and suggestions”, and “Made decisions that were based only on his/her decisions” (reverse-scored), “Encouraged group members to exchange information with one another”, “Helped the group focus on their goals”, and “Paid attention to the group’s efforts” (coefficient alpha $\hat{\alpha}$ = 0.77, categorical omega $\widehat{\omega_C}$ = 0.86).

Finally, we also include “information surfacing” as a behavioral measure of leader effectiveness. Unbeknownst to participants, the *PB Technologies* group decision-making task included hidden, asymmetric information about the CFO candidates (i.e., each group member held some unique information about the candidates that other group members did not know). Because the group leader was responsible for guiding the group discussion and helping the group come to a final agreement, an especially effective leader would help the group surface more pieces of information (out of 22 total). After the task, participants were given a list of 22 pieces of information (e.g., “Has a habit of being late to meetings”) and asked how many of these pieces of information they did not hear. We reversed this score into a total score of information surfacing (ranging from 0 to 22 pieces of information surfaced).

Results

Table S5 provides demographic data on our obtained sample versus the most recent U.S. Census data. Table S6 reports basic descriptive statistics and correlations. For a nationally representative sample participating in a group decision-making task with randomly assigned leaders (*N* = 305 ratings of 101 group leaders), we examined whether the group leader’s ambition predicted member-rated leadership effectiveness. In line with prior studies, we find moderate evidence *against* a relationship between ambition and third-party ratings of effectiveness, $\hat{\beta}$ = -0.07, 95% CI [-0.22, 0.07], *p* = .312, moderate BF$_{null}$ = 3.99. In other words, the data are about 4 times as likely under a null model (which does not include ambition) than the alternative (which does include ambition).

As our first robustness check, we examined the relationship between a group leader’s motivation to lead and member-rated leadership effectiveness. We again find moderate evidence *against* a relationship between motivation to lead and third-party ratings of effectiveness, $\hat{\beta}$ = 0.00, 95% CI [-0.17, 0.16], *p* = .979, moderate BF$_{null}$ = 6.40; that is, the data are over 6 times as likely under a null model (which does not include motivation to lead) than the alternative.

As our second robustness check, we examined the relationship between a group leader's ambition and member-rated empowering leader behavior. In line with prior findings, we find moderate evidence *against* a relationship between ambition and third-party ratings of empowering leader behavior, $\hat{\beta}$ = -0.06, 95% CI [-0.17, 0.04], *p* = .258, moderate BF$_{null}$ = 4.46.

As our third robustness check, we examined the relationship between a group leader's motivation to lead and member-rated empowering leader behavior. We again find moderate evidence *against* a relationship between motivation to lead and third-party ratings of empowering leader behavior, $\hat{\beta}$ = 0.00, 95% CI [-0.13, 0.12], *p* = .940, moderate BF$_{null}$ = 8.07.

Finally, we assess the relationship between ambition and effective behavioral outcomes. That is, are ambitious leaders able to surface more hidden pieces of information when guiding a group discussion? In line with our prior findings, we find moderate evidence *against* a relationship between ambition and effectiveness at surfacing information, $\hat{\beta}$ = -0.16, 95% CI [-0.55, 0.24], *p* = .436, moderate BF$_{null}$ = 4.78. Lastly, we examine the relationship between motivation to lead and information surfacing. Again, we find moderate evidence *against* a relationship between motivation to lead and information surfacing, $\hat{\beta}$ = -0.09, 95% CI [-0.54, 0.36], *p* = .693, moderate BF$_{null}$ = 5.81.

Exploratory Analysis of Gender Moderation

Once again, we explore whether the relationship between ambition and leadership evaluations is moderated by gender. We ran mixed effects models predicting effectiveness ratings from ambition, gender, an interaction effect (β_3) between ambition and gender, and random intercepts for leaders and survey items.

We find moderate evidence (3 < BF < 10) that the relationship between ambition and third-party ratings of leadership effectiveness is *not moderated by gender*, $\hat{\beta}_3$ = 0.05, 95% CI [-0.25, 0.36], *p* = .724, moderate BF$_{null}$ = 3.44. For our second measure of effectiveness, we again find moderate evidence (3 < BF < 10) that the relationship between ambition and third-party ratings of empowering leader behavior is *not moderated by gender*, $\hat{\beta}_3$ = -0.02, 95% CI [-0.25, 0.20], *p* = .852, moderate BF$_{null}$ = 4.17.

For our third measure of effectiveness, we find anecdotal evidence (1 < BF < 3) that the relationship between ambition and information surfacing is *not moderated by gender*, $\hat{\beta}_3$ = -0.34, 95% CI [-1.17, 0.49], *p* = .426, anecdotal BF$_{null}$ = 2.58.

Conclusion

Consistent with the results of prior studies, Supplemental Study 2 finds evidence against a relationship between ambition and third-party ratings of leader effectiveness. Using a nationally representative sample and a controlled leadership simulation, more ambitious individuals were rated as no more effective in a randomly assigned leadership role than less ambitious individuals. Notably, we find support for the null hypothesis across two predictors (ambition and motivation to lead) and three outcomes (leader effectiveness, empowering leader behavior, and information surfacing).

Acknowledgements

This supplement was created using R (Version 4.3.3; 26) and the R-packages *BayesFactor* (Version 0.9.12.4.6; 3), *coda* (Version 0.19.4.1; 27), *dplyr* (Version 1.1.4; 28), *ggplot2* (Version 3.5.1; 29), *here* (Version 1.0.1; 30), *knitr* (Version 1.45; 31), *lme4* (Version 1.1.35.3; 32), *lmerTest* (Version 3.1.3; 33), *Matrix* (Version 1.6.5; 34), *papaja* (Version 0.1.2.9000; 35), *purrr* (Version 1.0.2; 36), *readr* (Version 2.1.4; 37), *tibble* (Version 3.2.1; 38), *tidyr* (Version 1.3.1; 39), and *tinylabels* (Version 0.2.4; 40).

References

1. J. S. Ashby, I. Schoon, Career success: The role of teenage career aspirations, ambition value and gender in predicting adult social status and earnings. *Journal of Vocational Behavior* **77**, 350–360 (2010).
2. R. Neel, D. T. Kenrick, A. E. White, S. L. Neuberg, Individual differences in fundamental social motives. *Journal of Personality and Social Psychology* **110**, 887–907 (2016).
3. R. D. Morey, J. N. Rouder, *BayesFactor: Computation of bayes factors for common designs* (2022).
4. A. M. Stefan, F. Schönbrodt, N. J. Evans, E.-J. Wagenmakers, Efficiency in Sequential Testing: Comparing the Sequential Probability Ratio Test and the Sequential Bayes Factor Test (2020) https:/doi.org/10.31234/osf.io/ry4fw (November 29, 2022).
5. D. C. Funder, D. J. Ozer, Evaluating Effect Size in Psychological Research: Sense and Nonsense. *Advances in Methods and Practices in Psychological Science* **2**, 156–168 (2019).
6. G. E. Gignac, E. T. Szodorai, Effect size guidelines for individual differences researchers. *Personality and Individual Differences* **102**, 74–78 (2016).
7. T. A. Judge, J. E. Bono, R. Ilies, M. W. Gerhardt, Personality and leadership: A qualitative and quantitative review. *Journal of Applied Psychology* **87**, 765–780 (2002).
8. S. Andraszewicz, *et al.*, An introduction to Bayesian hypothesis testing for management research. *Journal of Management* **41**, 521–543 (2015).
9. C. J. Soto, O. P. John, Short and extra-short forms of the Big Five Inventory–2: The BFI-2-S and BFI-2-XS. *Journal of Research in Personality* **68**, 69–81 (2017).
10. K. Kelley, S. Pornprasertmanit, Confidence intervals for population reliability coefficients: Evaluation of methods, recommendations, and software for composite measures. *Psychological Methods* **21**, 69–92 (2016).
11. A. Ma, A. S. Rosette, C. Z. Koval, Reconciling female agentic advantage and disadvantage with the CADDIS measure of agency. *Journal of Applied Psychology* **107**, 2115–2148 (2022).
12. S. Humberg, *et al.*, Is accurate, positive, or inflated self-perception most advantageous for psychological adjustment? A competitive test of key hypotheses. *Journal of Personality and Social Psychology* **116**, 835–859 (2019).
13. O. Epitropaki, R. Martin, Implicit leadership theories in applied settings: Factor structure, generalizability, and stability over time. *The Journal of Applied Psychology* **89**, 293–310 (2004).
14. R. G. Lord, R. J. Foti, C. L. de Vader, A test of leadership categorization theory: Internal structure, information processing, and leadership perceptions. *Organizational Behavior & Human Performance* **34**, 343–378 (1984).
15. L. R. Offermann, M. R. Coats, Implicit theories of leadership: Stability and change over two decades. *The Leadership Quarterly* **29**, 513–522 (2018).
16. L. R. Offermann, J. K. Kennedy, P. W. Wirtz, Implicit leadership theories: Content, structure, and generalizability. *The Leadership Quarterly* **5**, 43–58 (1994).
17. S. V. Marinova, H. Moon, D. Kamdar, Getting ahead or getting along? The two-facet conceptualization of conscientiousness and leadership emergence. *Organization Science* **24**, 1257–1276 (2013).
18. Y. Kalish, G. Luria, Traits and time in leadership emergence: A longitudinal study. *The Leadership Quarterly* **32**, 101443 (2021).
19. J. Vergauwe, B. Wille, J. Hofmans, R. B. Kaiser, F. De Fruyt, The double-edged sword of leader charisma: Understanding the curvilinear relationship between charismatic personality and leader effectiveness. *Journal of Personality and Social Psychology* **114**, 110–130 (2018).
20. R. L. Schaumberg, F. J. Flynn, Uneasy lies the head that wears the crown: The link between guilt proneness and leadership. *Journal of Personality and Social Psychology* **103**, 327–342 (2012).

21. S. D. Gosling, P. J. Rentfrow, W. B. Swann, A very brief measure of the Big-Five personality domains. *Journal of Research in Personality* **37**, 504–528 (2003).
22. T. R. Cohen, S. T. Wolf, A. T. Panter, C. A. Insko, Introducing the GASP scale: A new measure of guilt and shame proneness. *Journal of Personality and Social Psychology* **100**, 947–966 (2011).
23. J. T. Cheng, J. L. Tracy, J. Henrich, Pride, personality, and the evolutionary foundations of human social status. *Evolution and Human Behavior* **31**, 334–347 (2010).
24. K.-Y. Chan, F. Drasgow, Toward a theory of individual differences and leadership: Understanding the motivation to lead. *Journal of Applied Psychology* **86**, 481–498 (2001).
25. J. A. Arnold, S. Arad, J. A. Rhoades, F. Drasgow, The Empowering Leadership Questionnaire: The Construction and Validation of a New Scale for Measuring Leader Behaviors. *Journal of Organizational Behavior* **21**, 249–269 (2000).
26. R Core Team, *R: A language and environment for statistical computing* (R Foundation for Statistical Computing, 2023).
27. M. Plummer, N. Best, K. Cowles, K. Vines, CODA: Convergence diagnosis and output analysis for MCMC. *R News* **6**, 7–11 (2006).
28. H. Wickham, R. François, L. Henry, K. Müller, D. Vaughan, *Dplyr: A grammar of data manipulation* (2023).
29. H. Wickham, *ggplot2: Elegant graphics for data analysis* (Springer-Verlag New York, 2016).
30. K. Müller, *Here: A simpler way to find your files* (2020).
31. Y. Xie, *Dynamic documents with R and knitr*, 2nd Ed. (Chapman; Hall/CRC, 2015).
32. D. Bates, M. Mächler, B. Bolker, S. Walker, Fitting linear mixed-effects models using lme4. *Journal of Statistical Software* **67**, 1–48 (2015).
33. A. Kuznetsova, P. B. Brockhoff, R. H. B. Christensen, lmerTest package: Tests in linear mixed effects models. *Journal of Statistical Software* **82**, 1–26 (2017).
34. D. Bates, M. Maechler, M. Jagan, *Matrix: Sparse and dense matrix classes and methods* (2023).
35. F. Aust, M. Barth, *papaja: Prepare reproducible APA journal articles with R Markdown* (2022).
36. H. Wickham, L. Henry, *Purrr: Functional programming tools* (2023).
37. H. Wickham, J. Hester, J. Bryan, *Readr: Read rectangular text data* (2023).
38. K. Müller, H. Wickham, *Tibble: Simple data frames* (2023).
39. H. Wickham, D. Vaughan, M. Girlich, *Tidyr: Tidy messy data* (2023).
40. M. Barth, *tinylabels: Lightweight variable labels* (2023).

Figures and Tables

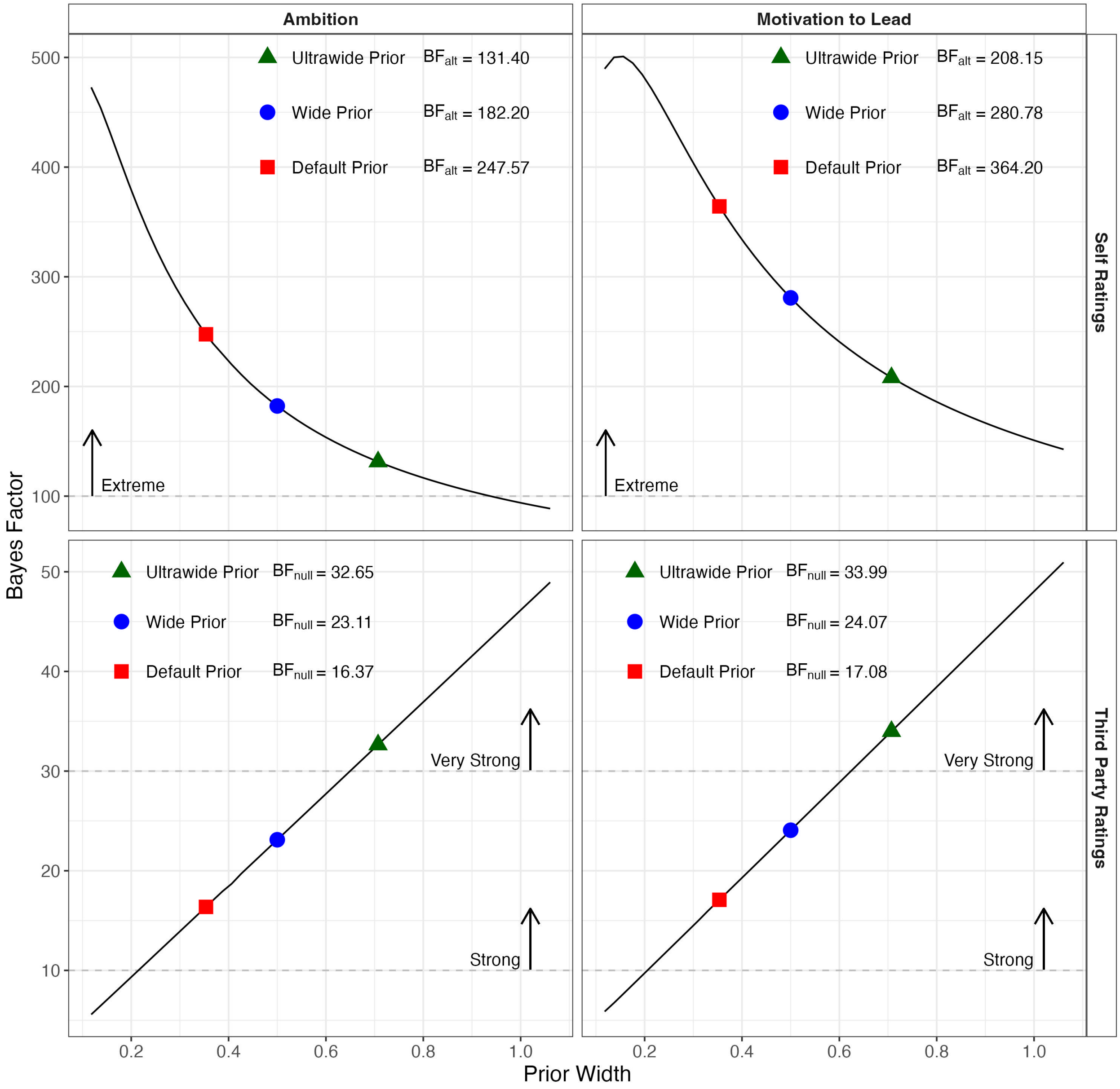

Figure S1. Bayes Factor robustness plot expressing the evidence *for* a relationship between ambition and self-evaluations of overall effectiveness in the leadership role (BF$_{alt}$), and the evidence *against* a relationship between ambition and third-party evaluations of overall effectiveness in the leadership role (BF$_{null}$), as a function of prior width. The prior assumes the slope is normally distributed with $\beta \sim N(0, g\sigma^2(X'X)^{-1})$, where g is Inverse Gamma distributed, $g \sim IG(1/2, r/2)$, and the default ("medium") prior sets $r = \sqrt{2}/4$, the wide prior sets $r = 1/2$, and the ultrawide prior sets $r = \sqrt{2}/2$. The evidence for our hypotheses is stable across the full range of prior distributions, suggesting that the analysis is robust.

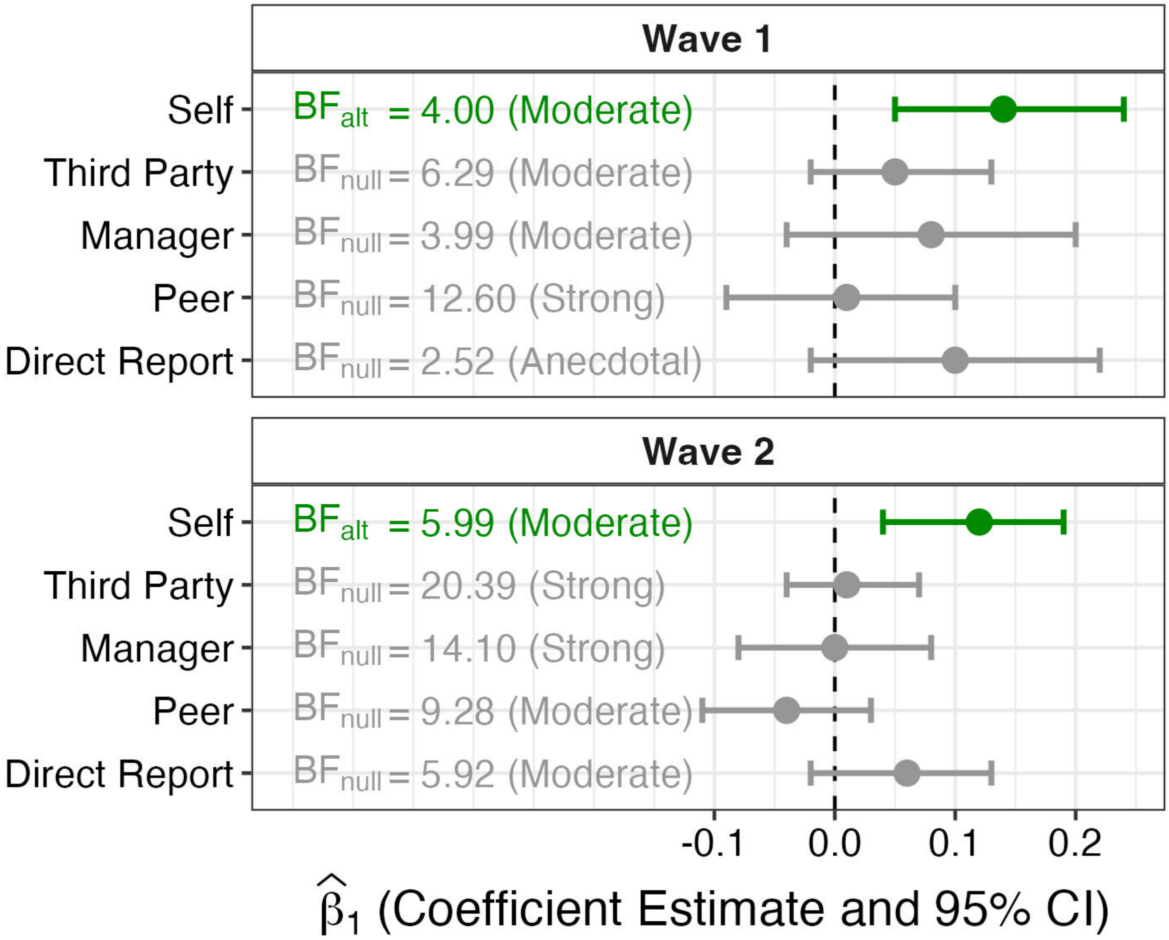

Figure S2. The relationship between ambition and leadership ratings for three measures of ambition, five rater roles, and two waves of data collection. We ran mixed effects models predicting effectiveness ratings from ambition as well as random intercepts for leaders and effectiveness items, and obtained the coefficient estimates for ambition ($\hat{\beta}_1$). The color of the bar indicates the significance of $\hat{\beta}_1$ (green bars indicate a rejection of the null; gray bars indicate a failure to reject the null). The Bayes Factors express the strength of the evidence: BF$_{alt}$ expresses the strength of the evidence for a full model (which includes a fixed effect for ambition), whereas BF$_{null}$ expresses the strength of the evidence for a null model (which does not include a fixed effect for ambition). Note that BF$_{alt}$ = 1 / BF$_{null}$. The color of the text indicates the direction of the evidence (green text indicates support for the full model; gray text indicates support for the null model).

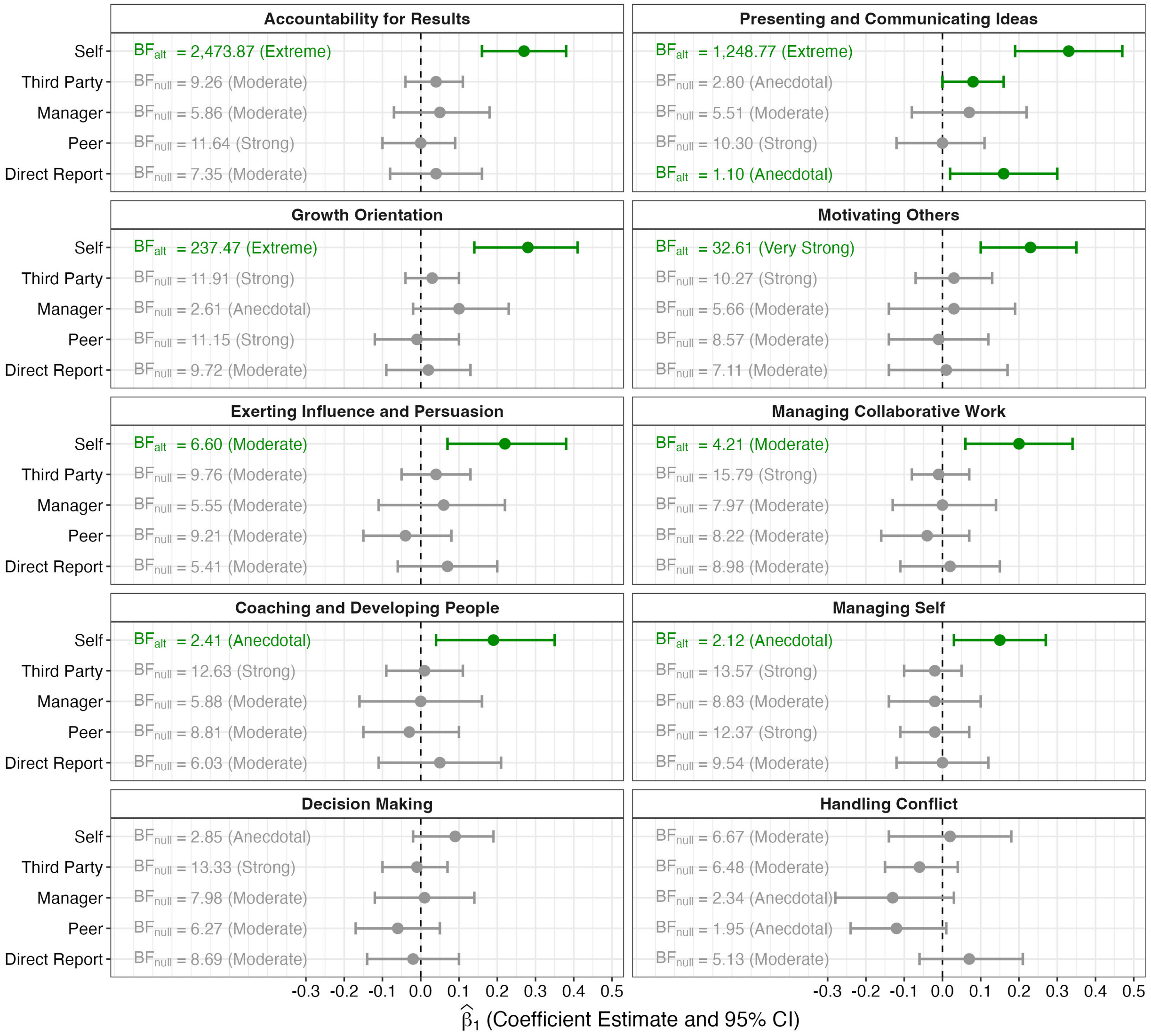

Figure S3. The relationship between motivation to lead and leadership ratings for ten leadership competencies and five rater roles. We ran mixed effects models predicting effectiveness ratings from motivation to lead as well as random intercepts for leaders and effectiveness items, and obtained the coefficient estimates for motivation to lead ($\hat{\beta}_1$). The color of the bar indicates the significance of $\hat{\beta}_1$ (green bars indicate a rejection of the null; gray bars indicate a failure to reject the null). The Bayes Factors express the strength of the evidence: BF_{alt} expresses the strength of the evidence for a full model (which includes a fixed effect for motivation to lead), whereas BF_{null} expresses the strength of the evidence for a null model (which does not include a fixed effect for motivation to lead). Note that $BF_{alt} = 1 / BF_{null}$. The color of the text indicates the direction of the evidence (green text indicates support for the full model; gray text indicates support for the null model).

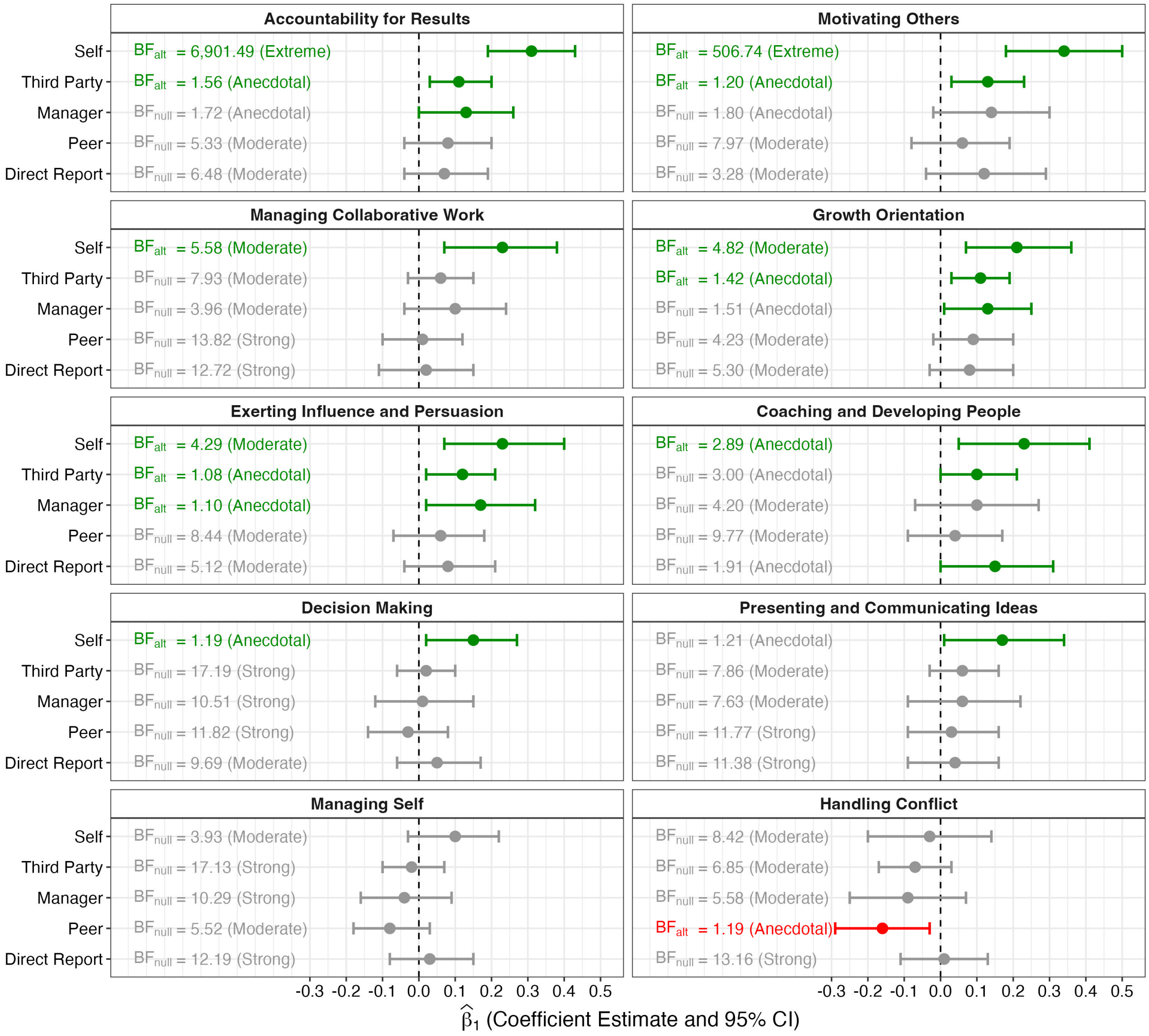

Figure S4. The relationship between drive to achieve and leadership ratings for ten leadership competencies and five rater roles. We ran mixed effects models predicting effectiveness ratings from drive to achieve as well as random intercepts for leaders and effectiveness items, and obtained the coefficient estimates for drive to achieve ($\hat{\beta}_1$). The color of the bar indicates the significance of $\hat{\beta}_1$ (green bars indicate a rejection of the null; gray bars indicate a failure to reject the null). The Bayes Factors express the strength of the evidence: BF$_{alt}$ expresses the strength of the evidence for a full model (which includes a fixed effect for drive to achieve), whereas BF$_{null}$ expresses the strength of the evidence for a null model (which does not include a fixed effect for drive to achieve). Note that BF$_{alt}$ = 1 / BF$_{null}$. The color of the text indicates the direction of the evidence (green text indicates support for the full model; gray text indicates support for the null model).

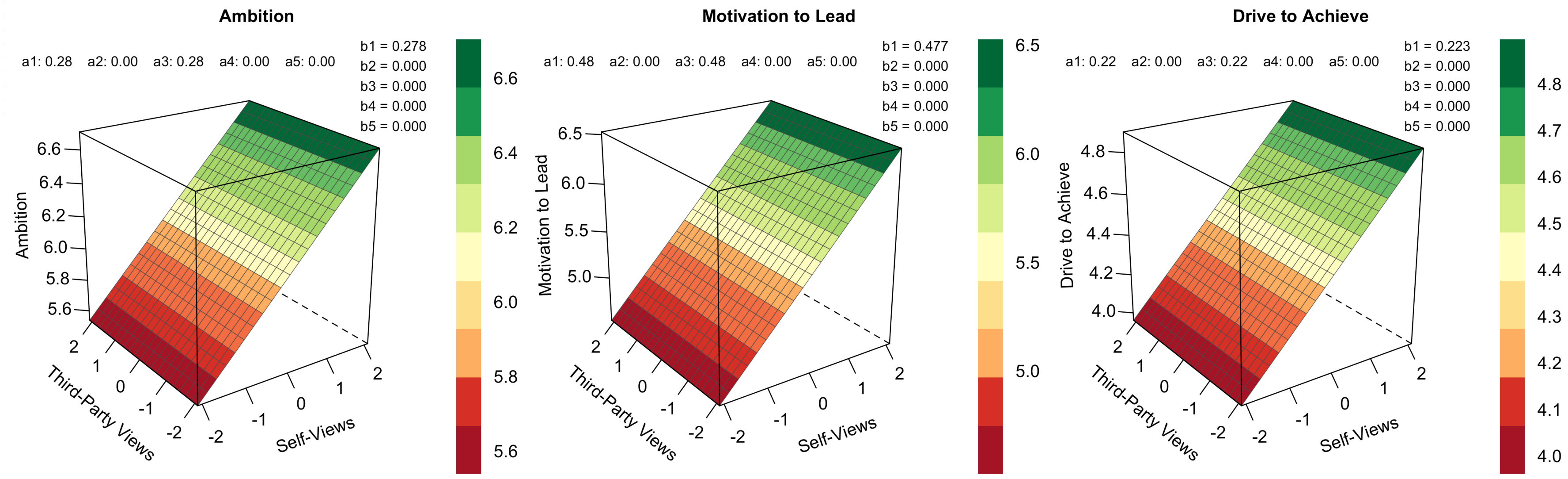

Figure S5. Response Surface Analyses. Self-views and third-party views refer to self-ratings and third-party ratings of leadership effectiveness, respectively. All three Response Surface Analyses provide evidence for the Uncorroborated Positive Self-Views model, which posits a positive relationship between ambition and self-views when controlling for third-party perceptions (i.e., $\beta_1 > 0$) and constrains the relationship between ambition and third-party perceptions to zero when controlling for self-views (i.e., $\beta_2 = 0$).

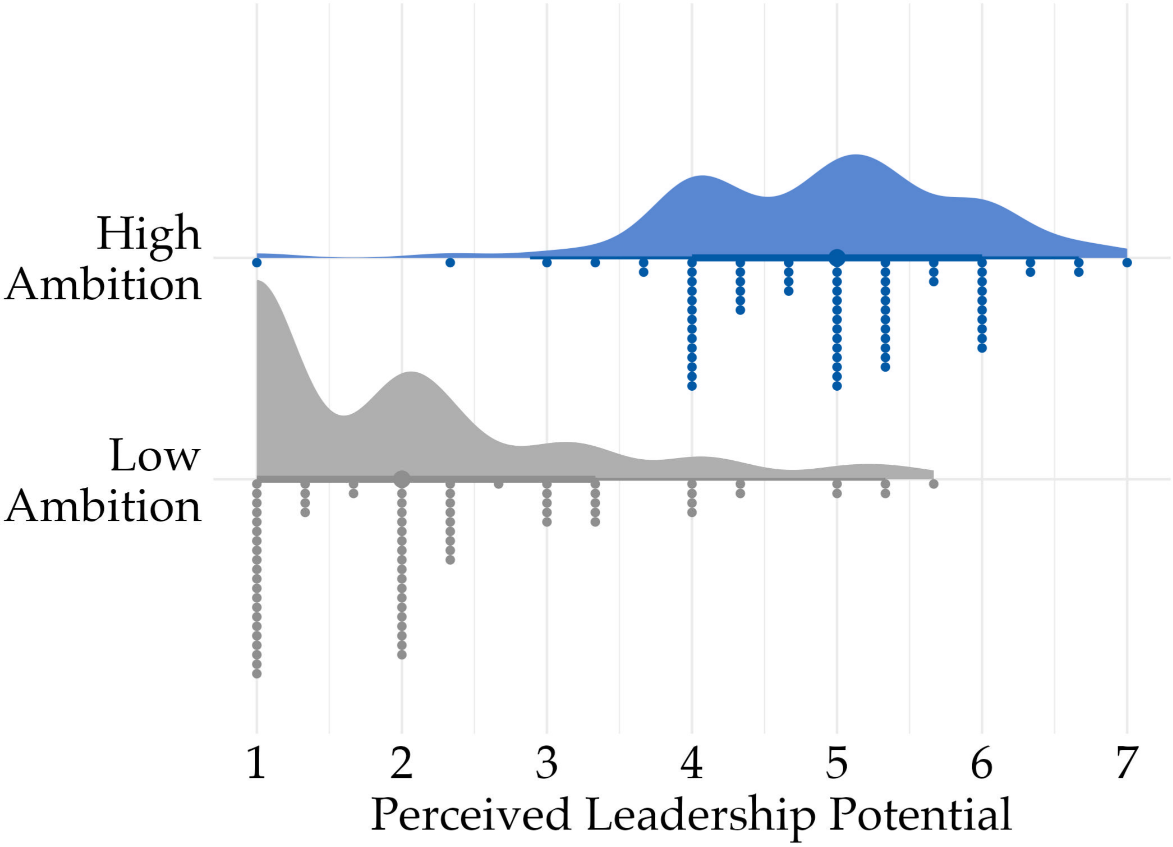

Figure S6. Perceived leadership potential as a function of experimental condition (high or low ambition) in Pilot Study 1. Ambition has a strong, positive effect on expectations of leader effectiveness, $\hat{\beta} = 2.83$, 95% CI [2.27, 2.99], $t(149) = 14.33$, $r = .76$, 95% CI [.68, .82], Cohen’s $d = 2.34$, $p < .001$.

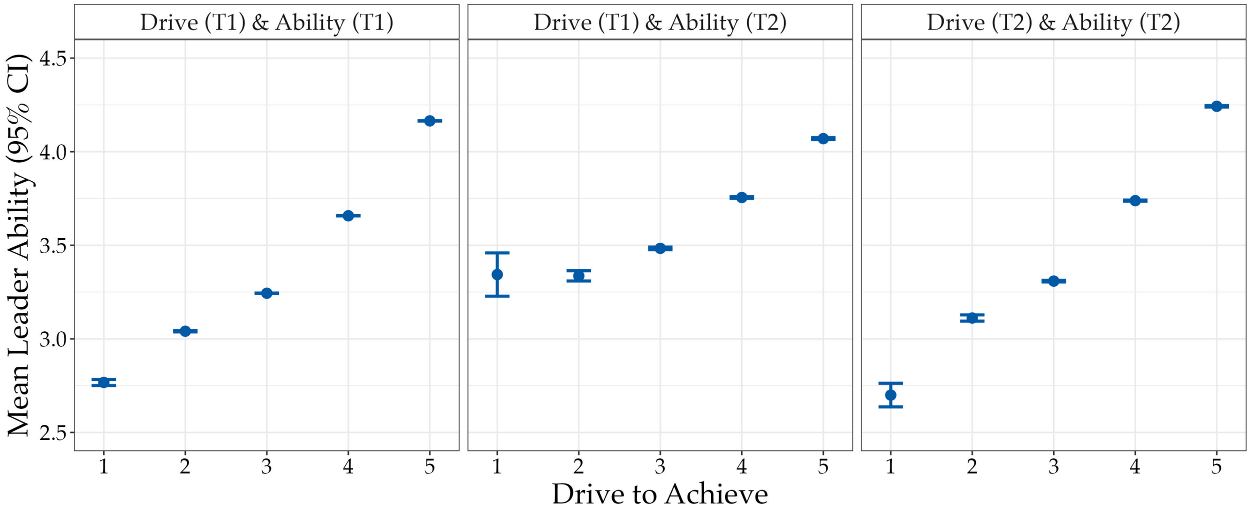

Figure S7. Average self-ratings of leadership ability (T1 and T2) as a function of drive to achieve (T1 and T2), supplemented with 95% CIs. We find a strong relationship between drive to achieve and self-ratings of leadership ability across all three preregistered tests.

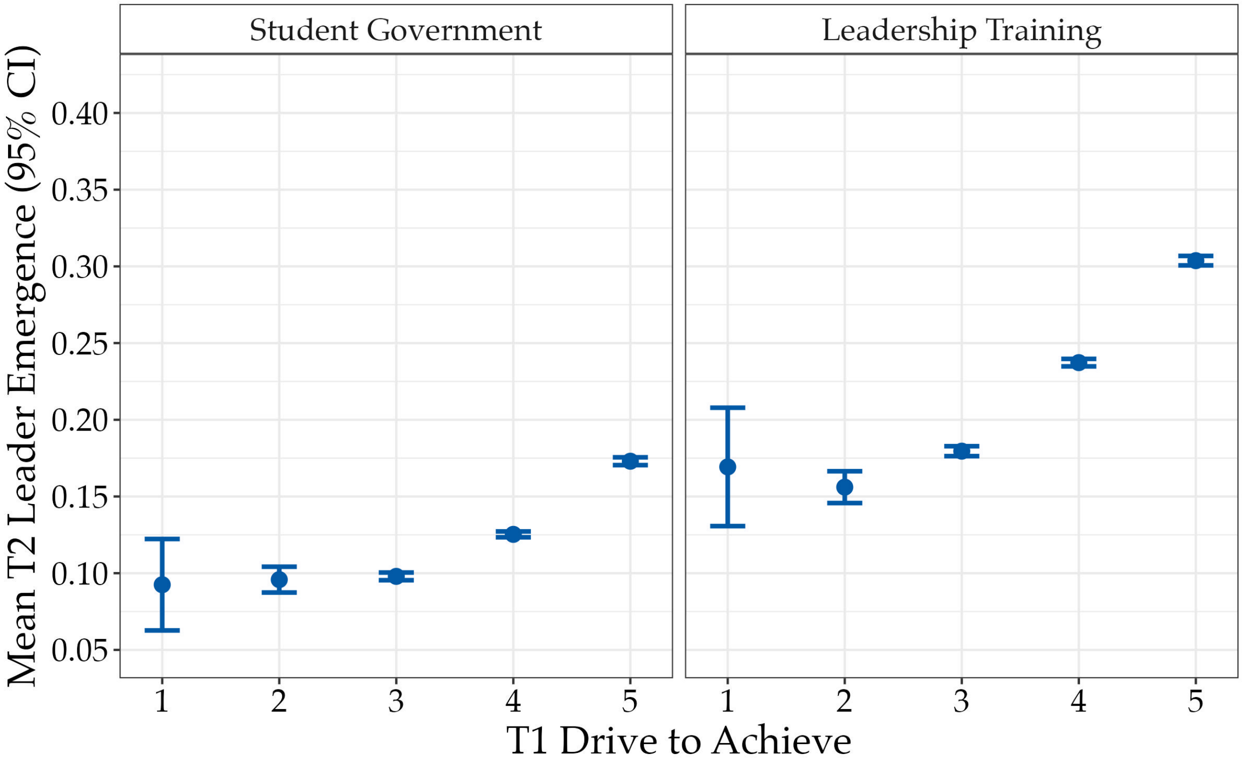

Figure S8. Average leader emergence rates (i.e., T2 participation rates in student government and leadership training) as a function of T1 drive to achieve, supplemented with 95% CIs. We find a strong relationship between drive to achieve and leader emergence across both preregistered tests.

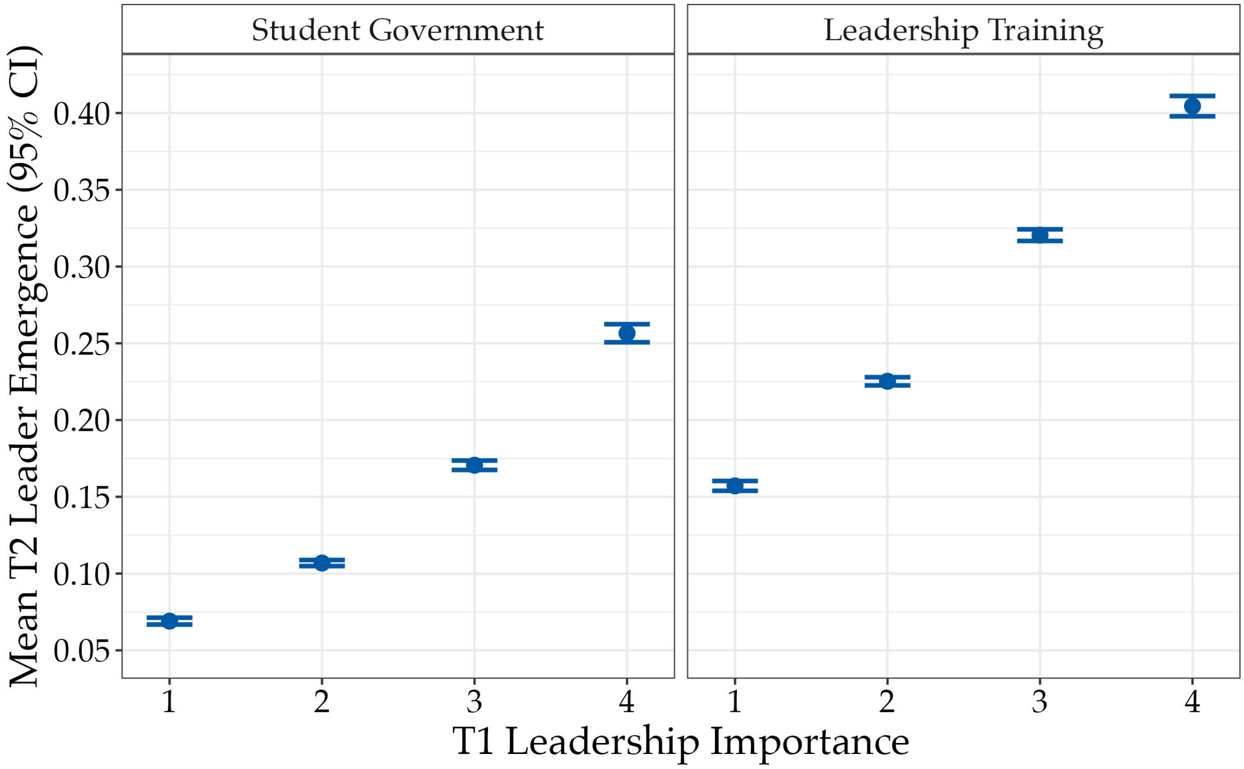

Figure S9. Average leader emergence rates (i.e., T2 participation rates in student government and leadership training) as a function of T1 importance of becoming a community leader, supplemented with 95% CIs. We find a strong relationship between leadership importance and leader emergence across both preregistered tests.

Table S1. Means, standard deviations, and correlations (Wave 1)

Variable	*M*	*SD*	1	2	3	4	5	6	7	8
1. Age	40.58	7.31								
2. Gender (1 = female)	0.36	0.48	-.09							
3. Ambition	6.13	0.80	-.15	-.00						
4. Motivation to lead	5.44	0.85	.01	.03	.32**					
5. Self ratings	5.50	0.54	.05	.19*	.21**	.31**				
6. Third-party ratings	5.85	0.40	.03	-.05	.10	.02	.18*			
7. Manager ratings	5.72	0.62	-.05	.03	.11	.03	.21**	.60**		
8. Peer ratings	5.85	0.53	-.04	.01	-.00	-.06	.06	.76**	.26**	
9. Subordinate ratings	5.95	0.60	.12	-.06	.14	.07	.24**	.66**	.17*	.17*

Note. M and *SD* are used to represent mean and standard deviation, respectively. * indicates $p < .05$. ** indicates $p < .01$.

Table S2. Means, standard deviations, and correlations (Wave 2)

Variable	*M*	*SD*	1	2	3	4	5	6	7	8	9	10
1. Age	42.09	6.98										
2. Gender (1 = female)	0.29	0.45	-.17*									
3. Ambition	6.02	0.85	-.02	-.01								
4. Drive to achieve	4.38	0.62	.11	-.04	.22**							
5. Self-rated leader ability	4.22	0.65	.09	-.04	.17**	.42**						
6. Self ratings	5.47	0.57	.09	.04	.18**	.21**	.31**					
7. Third party ratings	5.92	0.41	-.11	.01	.02	.11	.18**	.23**				
8. Manager ratings	5.78	0.57	-.02	-.02	.00	.09	.11	.19**	.60**			
9. Peer ratings	5.85	0.52	-.09	-.01	-.06	.02	.10	.18**	.82**	.36**		
10. Subordinate ratings	6.08	0.50	-.12	.12	.09	.09	.14*	.18**	.80**	.25**	.41**	
11. Extraversion	3.86	0.64	.03	.04	.21**	.27**	.38**	.28**	.08	.02	.05	.07

Note. M and *SD* are used to represent mean and standard deviation, respectively. * indicates $p < .05$. ** indicates $p < .01$.

Table S3. Means, standard deviations, and correlations (Pilot Study 2)

Variable	*M*	*SD*	1	2	3	4	5	6	7	8
1. Gender (1 = female)	0.63	0.48								
2. T1 Drive to achieve	4.08	0.78	-.01**							
3. T1 Community leader	2.23	0.90	-.04**	.24**						
4. T1 Leader ability	3.74	0.88	-.09**	.37**	.41**					
5. T2 Drive to achieve	4.04	0.82	-.01**	.41**	.18**	.24**				
6. T2 Community leader	2.22	0.93	-.07**	.18**	.43**	.29**	.24**			
7. T2 Leader ability	3.78	0.86	-.16**	.26**	.30**	.51**	.42**	.39**		
8. T2 Student government	0.12	0.32	-.02**	.08**	.16**	.15**	.08**	.17**	.17**	
9. T2 Leader training	0.22	0.42	.01**	.11**	.17**	.18**	.11**	.23**	.25**	.27**

Note. M and *SD* are used to represent mean and standard deviation, respectively. * $p < .05$. ** $p < .01$.

Table S4. Means, standard deviations, and correlations (Supplemental Study 1)

Variable	*M*	*SD*	1	2	3	4	5	6	7	8	9
1. Age	27.48	2.13									
2. Gender (1 = female)	0.46	0.50	-.21**								
3. Ambition	5.95	0.73	.04	-.08							
4. Status motive	5.87	0.83	-.14	.03	.56**						
5. Extraversion	4.42	1.74	-.08	.01	.16*	.25**					
6. Agreeableness	5.10	1.09	-.06	.16*	-.09	.03	.04				
7. Conscientiousness	5.67	1.06	-.04	-.03	.18*	.21**	.07	.07			
8. Neuroticism	3.02	1.37	-.08	.27**	-.11	-.08	-.11	-.17*	-.24**		
9. Openness	5.10	1.16	.10	-.03	.05	.09	.19*	-.11	.11	-.11	
10. Effectiveness	5.26	0.88	-.10	.07	-.08	.06	.19*	.13	.02	-.00	.12

Note. M and *SD* are used to represent mean and standard deviation, respectively. * indicates p < .05. ** indicates p < .01.

Table S5. Sample Characteristics vs. U.S. Census Data (Supplemental Study 2)

Category	Study Sample	U.S. Census
**Gender**		
Female	52.8%	50.3%
Male	45.9%	48.7%
Non-binary	1.2%	1.0%
**Age**		
18-24 years old	14.3%	12.8%
25-34 years old	17.8%	17.7%
35-44 years old	21.2%	16.5%
45-54 years old	17.8%	17.7%
55-64 years old	18.8%	16.4%
65+ years old	10.1%	18.9%
**Location**		
Northeast	21.0%	17.6%
South	33.6%	37.6%
Midwest	21.2%	21.2%
West	24.2%	23.6%
**Race/Ethnicity**		
Asian/Pacific Islander	6.4%	5.9%
Black/African American	16.3%	13.4%
European American/White	65.4%	60.1%
Hispanic/Latino**	10.4%	18.2%
Other	0.09%	2.4%
**Income**		
Less than $25,000	14.7%	22.3%
$25,000 to $34,999	11.5%	9.9%
$35,000 to $49,999	15.0%	13.2%
$50,000 to $74,999	22.4%	17.8%
$75,000 to $99,999	14.7%	12.2%
$100,000 to $149,999	14.5%	13.5%
$150,000 to $199,999	4.7%	5.4%
$200,000 or more	2.5%	5.7%

Note. Income data were provided by the third-party recruitment firm's participant database. Race/ethnicity and location were collected in the pre-screen administered by the recruitment firm. **The percentage of Hispanic participants reflected in our prescreen data may be underestimated because, unlike the U.S. Census, we did not administer a second question solely about Hispanic/Latino descent. Using the recruitment firm's panel database where they do administer a second question about Hispanic/Latino descent, they estimate this percentage to be closer to 14.1% in our sample.

Table S6. Means, standard deviations, and correlations (Supplemental Study 2)

Variable	*M*	*SD*	1	2	3	4	5	6
1. Gender (1 = female)	0.53	0.50						
2. Age	44.67	15.64	.18					
3. Ambition	5.18	1.46	-.19	-.23*				
4. Motivation to lead	4.37	1.29	-.17	-.12	.72**			
5. Effectiveness	5.30	1.07	.02	-.06	-.10	-.00		
6. Empowering behavior	5.72	0.80	.01	-.04	-.11	-.01	.79**	
7. Information surfacing	14.91	2.94	.10	-.12	-.08	-.05	.22*	.16

Note. *M* and *SD* are used to represent mean and standard deviation, respectively. * indicates $p < .05$. ** indicates $p < .01$.
